# Characterization of the Methanomicrobial Archaeal RNase Zs for Processing the CCA-Containing tRNA Precursors

**DOI:** 10.3389/fmicb.2020.01851

**Published:** 2020-08-25

**Authors:** Xiaoyan Wang, Xien Gu, Jie Li, Lei Yue, Defeng Li, Xiuzhu Dong

**Affiliations:** ^1^Department of Biochemistry and Molecular Biology, Institute of Basic Medical Sciences, College of Basic Medicine, Hubei University of Medicine, Shiyan, China; ^2^Hubei Key Laboratory of Embryonic Stem Cell Research, Hubei University of Medicine, Shiyan, China; ^3^State Key Laboratory of Microbial Resources, Institute of Microbiology, Chinese Academy of Sciences, Beijing, China; ^4^State Key Laboratory of Microbial Resources, University of Chinese Academy of Sciences, Beijing, China

**Keywords:** aRNase Z, precursor tRNA, 3′ end processing, tRNA maturation, CCA motif, methanomicrobial archaea

## Abstract

RNase Z is a widely distributed and usually essential endoribonuclease involved in the 3′-end maturation of transfer RNAs (tRNAs). A CCA triplet that is needed for tRNA aminoacylation in protein translation is added by a nucleotidyl-transferase after the 3′-end processing by RNase Z. However, a considerable proportion of the archaeal pre-tRNAs genetically encode a CCA motif, while the enzymatic characteristics of the archaeal RNase (aRNase) Zs in processing CCA-containing pre-tRNAs remain unclear. This study intensively characterized two methanomicrobial aRNase Zs, the *Methanolobus psychrophilus* mpy-RNase Z and the *Methanococcus maripaludis* mmp-RNase Z, particularly focusing on the properties of processing the CCA-containing pre-tRNAs, and in parallel comparison with a bacterial bsu-RNase Z from *Bacillus subtilis*. Kinetic analysis found that Co^2+^ supplementation enhanced the cleavage efficiency of mpy-RNase Z, mmp-RNase Z, and bsu-RNase Z for 1400-, 2990-, and 34-fold, respectively, and Co^2+^ is even more indispensable to the aRNase Zs than to bsu-RNase Z. Mg^2+^ also elevated the initial cleavage velocity (V_0_) of bsu-RNase Z for 60.5-fold. The two aRNase Zs exhibited indiscriminate efficiencies in processing CCA-containing vs. CCA-less pre-tRNAs. However, V_0_ of bsu-RNase Z was markedly reduced for 1520-fold by the CCA motif present in pre-tRNAs under Mg^2+^ supplementation, but only 5.8-fold reduced under Co^2+^ supplementation, suggesting Co^2+^ could ameliorate the CCA motif inhibition on bsu-RNase Z. By 3′-RACE, we determined that the aRNase Zs cleaved just downstream the discriminator nucleotide and the CCA triplet in CCA-less and CCA-containing pre-tRNAs, thus exposing the 3′-end for linking CCA and the genetically encoded CCA triplet, respectively. The aRNase Zs, but not bsu-RNase Z, were also able to process the intron-embedded archaeal pre-tRNAs, and even process pre-tRNAs that lack the D, T, or anticodon arm, but strictly required the acceptor stem. In summary, the two methanomicrobial aRNase Zs use cobalt as a metal ligand and process a broad spectrum of pre-tRNAs, and the characteristics would extend our understandings on aRNase Zs.

## Introduction

Transfer RNAs (tRNAs) carry specific amino acids to ribosome and decode messenger RNAs (mRNAs) through base-pairing between the anticodon sequence in tRNA and the corresponding codon in mRNA, and thus play a pivotal role in protein translation (Redko et al., 2007; Kirchner and Ignatova, 2015). All tRNAs are transcribed as precursors that have to be processed to the mature functional form. Maturation of tRNA precursors (pre-tRNAs) is accomplished through several processing steps, including removal of the 5′-leader and 3′-trailer sequences, excision of introns in eukaryotic and some archaeal pre-tRNAs, nucleotide modifications, and the addition of a CCA triplet at the 3′-end (Redko et al., 2007). The endoribonuclease RNase P performs the 5′-end processing (Guerriertakada et al., 1983; Holzmann et al., 2008), while the endoribonuclease RNase Z plays an essential role in removal of the 3′-trailer from pre-tRNAs, and the CCA triplet that is needed for tRNA aminoacylation is added by a nucleotidyl-transferase after the 3′-end processing by RNase Z (Cudny and Deutscher, 1986; Cudny et al., 1986).

The tRNA 3′-end maturation through an endoribonucleolytic processing was first discovered in eukaryotes (Nashimoto, 1997; Kunzmann et al., 1998; Mohan et al., 1999; Schiffer et al., 2001). The functional endoribonuclease, RNase Z, was first purified as a homogeneous protein from *Arabidopsis thaliana* (Schiffer et al., 2002), and then its orthologs have been widely characterized among bacteria (Pellegrini et al., 2003; Minagawa et al., 2004; Ceballos-Chavez and Vioque, 2005; Ezraty et al., 2005; Dutta and Deutscher, 2009), and some halophilic and thermophilic euryarchaea (Schierling et al., 2002; Spaeth et al., 2008). These investigations have demonstrated that RNase Z endonucleolytically hydrolyzes the phosphodiester bond directly downstream of the discriminator nucleotide, an unpaired nucleotide at the 3′-end of the acceptor stem, thus resulting in a processed tRNA with a 3′-hydroxyl group. These studies also found that the nucleotide type of the discriminator does not affect but cytidines present directly downstream it severely suppress the 3′-trailer removal efficiency of RNase Z. The *Bacillus subtilis*, and wheat RNase Zs, all poorly cleave a 3′-trailer sequence beginning with CC, CCA, or CCA with one or two extra nucleotides. So the CCA motif in pre-tRNA 3′-end has been identified as a general repressor of RNase Zs (Mohan et al., 1999; Pellegrini et al., 2003). Different from the eukaryotic tRNA genes, which do not encode the CCA motif in general, the archaeal and bacterial tRNA genes, with varying proportions, encode the CCA motif (Redko et al., 2007). Therefore, in bacteria, a different processing pathway, which involves concerted actions of the endoribonuclease RNase E and a handful of exonucleases, is employed to trim the 3′ extension downstream of the encoded CCA end (Li and Deutscher, 1996, 2002; Ow and Kushner, 2002; Wen et al., 2005). In some cases, RNase E cleavage directly generates the mature CCA terminus (Mohanty et al., 2016).

However, not all RNase Zs are generally inhibited by the CCA motif, such as the *Thermotoga maritima* RNase Z cleaves just downstream the CCA triplets in 45 tRNAs that carry the genetically encoded CCA (Minagawa et al., 2004), indicating that it could process the 3′-end of CCA-containing pre-tRNAs in one step. Moreover, the *Escherichia coli* RNase Z, previously misidentified as RNase BN, is able to remove the 3′-end of pre-tRNAs ending as CA, CU, CCU, or even CCA (Asha et al., 1983). Additionally, a mammalian RNase Z also exhibits a measurable activity of cleaving downstream the CCA motif (Nashimoto, 1997). However, whether aRNase Zs process the CCA-containing pre-tRNAs as CCA-less ones have not been thoroughly elucidated.

RNase Z belongs to the family of metal-dependent β-lactamases, a group of metalloproteins that possess a conserved structural β-lactamase domain but with highly divergent sequences and functions (Callebaut et al., 2002). The protein structures of RNase Zs from *B. subtilis* (Li de la Sierra-Gallay et al., 2005, 2006), *T. maritima* (Ishii et al., 2005), and *E. coli* (Kostelecky et al., 2006) all reveal a dimer of Zn^2+^-containing metallo-β-lactamase domains with a protruded flexible arm that involves in tRNA binding. However, although two Zn^2+^ ions were observed in the catalytic center of the bacterial RNase (bRNase) Zs (Ishii et al., 2005; Li de la Sierra-Gallay et al., 2005), addition of Zn^2+^ did not, but Co^2+^ elevated the activity of *E. coli* RNase Z (Asha et al., 1983). Moreover, Mn^2+^ also stimulates the 3′-end processing activity of RNase Zs from *T. maritima* (Minagawa et al., 2006), *A. thaliana* (Spath et al., 2007), *Haloferax volcanii*, and *Pyrococcus furiosus* (Spaeth et al., 2008). Mn^2+^ amendment even rescues the lost Mg^2+^-dependent activity of the *T. maritima* RNase Z catalytic mutants (Minagawa et al., 2006).

Methanomicrobial archaea are the most cultured and the most widely distributed archaeal representatives. They belong to the superphylum Euryarchaeota, one of the four major archaeal superphyla (Eme et al., 2017; Evans et al., 2019). However, the methanomicrobial aRNase Zs have not been intensively characterized. In this work, the *Methanolobus psychrophilus* mpy-RNase Z and *Methanococcus maripaludis* mmp-RNase Z were thoroughly investigated for the 3′-end processing activities in parallel comparison with a bRNase Z from *B. subtilis*, particularly focusing on the properties of processing CCA-containing pre-tRNAs. We found that Co^2+^ dramatically enhanced the catalytic efficiencies of both the archaeal and bRNase Zs for 1400-, 2990-, and 34-fold, respectively, and Co^2+^ appeared to be indispensable to the two aRNase Zs. The two methanomicrobial aRNase Zs indiscriminately processed CCA-containing and CCA-less pre-tRNAs, but CCA-motif severely suppressed the initial velocity of bsu-RNase Z for 1520-fold unless Co^2+^ supplementation. Moreover, the two aRNase Zs were capable of processing the intron-containing pre-tRNAs and the aberrant pre-tRNA that contains only the acceptor stem, but required a mature 5′-end for the 3′-end processing and regardless of the 3′ trailer lengths.

## Materials and Methods

### Strains, Culture Conditions, and Genomic DNA Extraction

*Methanococcus maripaludis* S2 and *M. psychrophilus* R15 were, respectively, grown in pre-reduced McF medium at 37°C (Sarmiento et al., 2011) and a defined mineral medium containing 20 mM trimethylamine at 18°C (Qi et al., 2017), and under a gas phase of N_2_/CO_2_ (80:20 v/v, 0.1 MPa). *E. coli* DH5α, Rosetta (DE3), and *B. subtilis* 168 were grown at 37°C in Luria–Bertani (LB) broth supplemented with ampicillin (100 μg/ml) or kanamycin (50 μg/ml) when required.

The genomic DNA of *M. psychrophilus* R15, *M. maripaludis* S2, and *B. subtilis* 168 was extracted and purified from the mid-exponential cultures using the TIANamp Bacteria DNA Kit (TIANGEN Biotech, Beijing, China) by following the manufacturer’s protocol. Purified DNA was quantified using a NanoPhotometer spectrophotometer (IMPLEN, Westlake Village, CA, United States), and DNA quality was checked by 2% agarose gel electrophoresis.

### Cloning and Protein Expression

The open reading frames *Mpsy_2804* encoding mpy-RNase Z, *mmp0906* encoding mmp-RNase Z, and *bsu23840* encoding bsu-RNase Z were amplified by polymerase chain reaction (PCR) using the respective genomic DNAs and cloned between the *Nco*I site and the His tag encoding site of plasmid pET28a (Novagen) *via* stepwise Gibson assembly using the ClonExpress MultiS One Step Cloning Kit (Vazyme). The expression plasmids produce recombinant protein with a C-terminal His_6_ tag. All cloned DNA sequences were verified by DNA sequencing, and the expression plasmids were transformed into *E. coli* Rosetta (DE3) competent cells. Transformants were cultured at 37°C in LB containing 50 μg⋅ml^–1^ kanamycin until OD_600_ at 0.6–0.8, followed by 16 h of induction with 0.1 mM isopropyl-β-D-thiogalactoside at 22°C. Cells were harvested by centrifugation at 5000 × *g* for 30 min at 4°C and then stored at −80°C until further analysis.

### Protein Purification

The three RNase Zs – mpy-RNase Z, mmp-RNase Z, and bsu-RNase Z – were purified using a similar method as previously described (Zheng et al., 2017). The harvested cells were resuspended in binding buffer [20 mM HEPES pH 7.5, 500 mM NaCl, 20 mM imidazole, and 5% (w/v) glycerol], lysed by sonication, and centrifuged at 10,000 × *g* for 60 min at 4°C. Then, supernatant was loaded on a HisTrap HP column (GE Healthcare) equilibrated with binding buffer and eluted with a linear gradient from 50 to 500 mM imidazole. The eluted protein was then dialyzed against buffer A [20 mM HEPES pH 7.5, 25 mM NaCl, 0.1 mM EDTA, and 5% (w/v) glycerol], loaded onto a HiTrap Q HP column (GE Healthcare), and eluted with a linear gradient of 100 mM to 1 M NaCl to remove the contaminative RNA and proteins. The eluted protein was validated to be RNA-free via measurement of the OD_260_ to OD_280_ ratio by a NanoDrop 2000 UV-Vis spectrophotometer (Thermo). Proteins with an OD_260_ to OD_280_ ratio below 0.6 were considered RNA-/DNA-free (Niedner et al., 2013). Finally, the purity of the RNA-free proteins was determined by SDS-PAGE, and the homogeneous protein was dialyzed against buffer B [20 mM HEPES pH 7.5, 150 mM NaCl, 5% (w/v) glycerol], concentrated using Amicon Ultrafra -30 concentrators (Millipore), flash-frozen, and stored at −80°C for biochemical assays. Protein concentration was determined using the Pierce BCA protein assay kit (Thermo Scientific).

### *In vitro* Transcription of Pre-tRNA Substrates

Through *in vitro* transcription using the forward primer containing a T7 RNA polymerase promoter sequence (Supplementary Table S1), the pre-tRNA substrates (Supplementary Table S2) were prepared. The DNA templates of pre-tRNA^mpy–Arg1^ and its variants that carry varying lengths of 5′ or 3′ extensions, pre-tRNA^mpy–Arg2(CCA)^, pre-tRNA^mpy–Arg3(intron)^, and pre-tRNA^mpy–Tyr(intron)^ were prepared by PCR amplification from the genomic DNA of *M. psychrophilus* R15 using the corresponding primer pairs (Supplementary Table S1). The templates of pre-tRNA^mmp–Arg1^ and its variants carrying varying lengths of 5′ or 3′ extensions, pre-tRNA^mmp–Arg2(CCA)^, pre-tRNA^bsu–trnI^, pre-tRNA^bsu–trnB(CCA)^, and pre-tRNA^bsu–trn62(CCA)^ were all prepared similarly. While the templates of pre-tRNA^mpy–Arg1^ and pre-tRNA^mmp–Arg1^ variants missing one mature tRNA part, either the D arm, anticodon arm, T arm, or acceptor stem, were synthesized by Sangon Biotech (Shanghai, China). Sequences of the pre-tRNAs used in this study are all listed in Supplementary Table S2. The amplified DNA templates were purified with a Wizard Gel and PCR Clean-UP System (Promega).

Next, the pre-tRNA substrates were produced by *in vitro* transcription as described previously (Zheng et al., 2017). *In vitro* transcriptions were conducted using the MEGAshortscript T7 Kit (Ambion) according to the manufacturer’s instructions, and the transcribed RNA products were further purified by 10% denatured PAGE containing 8 M urea using the ZR small-RNA TMPAGE Recovery Kit (ZYMO) and then quantified with a NanoPhotometer spectrophotometer (IMPLEN, Westlake Village, CA, United States).

### Nuclease Assays of RNase Zs

RNase Z activity was assayed in a 10 μl reaction mixture in 20 mM HEPES pH 7.5, 150 mM NaCl, and 5% glycerol in the absence or presence of 1 mM ZnCl_2_, CuCl_2_, MgCl_2_, or CoCl_2_, and incubated at 37°C for 30 min or indicated time. Generally, 1.4 pmol pre-tRNA was used in all assays, and the protein concentration is indicated in each figure or figure legend. Reactions were initiated by the addition of enzyme, incubated at 37°C for 5–45 min, and stopped by incubation with 0.5 μg/ml Proteinase K (Ambion) at 55°C for 15 min. After incubation, the reaction mixtures were mixed with formamide-containing dye (98% formamide, 5 mM EDTA, 0.025% bromophenol blue, 0.025% xylene cyanol, 0.025% SDS) and analyzed by 10% PAGE with 8 M urea. Oligoribonucleotides in lengths of 50, 80, and 169 nt were used as a molecular ladder to indicate the migration positions for RNA substrates and products. The urea-PAGE gels were stained by SYBR Gold for 10 min and then analyzed by fluorescence imaging with a Bio-Rad Gel doc XR+ (BIO-Rad).

Kinetic parameters of the three RNase Zs were determined by replicative quantification of the cleaving rate on various amounts (0.025–1 μM) of the CCA-less and CCA-containing pre-tRNAs under different conditions listed in Table 1 and Supplementary Figure S4. The reactions were performed at 37°C and the initial velocity (V_0_) was determined through quantifying the substrate residuals in the linear phase during the initial 5 min as that shown in Figure 3. The substrate residual contents were rigorously quantified using the Quantity One software for three independent quantifications of the non-overexposure gels, and the confidential sampling points were used in V_0_ and the Lineweaver–Burk plotting calculation. The Lineweaver–Burk plots of the three RNase Zs for each substrate are shown in Supplementary Figure S4. The kinetic parameters of K_m_, V_max_, and k_cat_ were obtained by fitting the data to the Michaelis-Menten equation.

**TABLE 1:** Kinetic parameters of mpy-, mmp-, and bsu-RNase Zs for pre-tRNA 3′-end processing.

Protein	Substrate	CoCl_2_	K_m_ (μM)	k_cat_ (min^–1^)	k_cat_/K_m_ (μM^–1^ min^–1^)
mpy-RNase Z	Pre-tRNA^mpy–Arg1^	−	0.6	0.0143	0.024
	Pre-tRNA^mpy–Arg1^	+	0.26	8.7	33.46
	Pre-tRNA^mpy–Arg2(CCA)^	+	0.43	12	27.91
mmp-RNase Z	Pre-tRNA^mmp–Arg1^	−	0.809	0.0045	0.0056
	Pre-tRNA^mmp–Arg1^	+	0.36	6.3	17.5
	Pre-tRNA^mmp–Arg2(CCA)^	+	0.25	13.5	54
bsu-RNase Z	Pre-tRNA^bsu–trnI^	−	0.87	0.84	0.957
	Pre-tRNA^bsu–trnI^	+	0.89	28.8	32.36
	Pre-tRNA^bsu–trn62^ ^(CCA)^	+	0.56	1.4	2.5

*A range of (0.025–1 μM) the genetically encoded CCA-lacking and CCA-containing pre-tRNA substrate concentrations were assayed for each tested RNase Z at 37°C in the absence (−) or presence (+) of 1 mM CoCl_2_. Initial velocity (V_0_) of RNase Z at each substrate concentration was determined through quantifying the substrate residuals in the linear phase during the initial 5 min similarly as that shown in Figure 3. The Lineweaver–Burk plots of the three RNase Zs for each substrate were shown in Supplementary Figure S4. The kinetic parameters of K_m_, V_max_, and k_cat_ were obtained by fitting the data to the Michaelis-Menten equation. Each set of values was from the averages of three replicates with a standard deviation of 3–15%.*

### Mapping of RNase Z Cleavage Sites

The cleavage sites in the CCA-less pre-tRNA^mmp–Arg1^ and CCA-containing pre-tRNA^mmp–Arg2(CCA)^ generated by the three RNase Zs were mapped by rapid amplification of cDNA 3′-ends (3′-RACE). The primary cleaved products were first spliced from 10% urea-PAGE, recovered, and purified using the ZR small-RNA PAGE Recovery Kit (ZYMO) and quantified with a NanoPhotometer spectrophotometer (IMPLEN, Westlake Village, CA, United States). 3′-RACE was performed as described previously (Zhang et al., 2009). Briefly, total RNA (10 μg) was ligated with 50 pmol 3′-adaptor linker (5′-(rApp)CTGTAGGCACCATCAAT–NH_2_-3′; NEB) through a 16 h incubation at 16°C with 20 U T4 RNA ligase (Ambion); 3′ linker-ligated RNA was recovered by isopropanol precipitation, and one aliquot of 4 μg was mixed with 100 pmol 3′R-RT-P (5′-ATTGATGGTGCCTACA G-3′, complementary to the 3′ RNA linker) by incubation at 65°C for 10 min and on ice for 2 min. This sample was used in the reverse transcription (RT) reaction using 200 U of SuperScript III reverse transcriptase (Invitrogen). After RT, gene-specific PCR was conducted using primers (Supplementary Table S1) complementary to the 5′-end of pre-tRNAs to obtain specific products. Specific PCR products were excised from a 2% agarose gel, cloned into pMD19-T (TaKaRa), and sequenced. The 3′-ends of the RNase Z cleavage products were defined as the nucleotide linked to the 3′-RACE linker.

## Results

### RNase Z Orthologs Are Widely Distributed in Archaea

**FIGURE 1:**
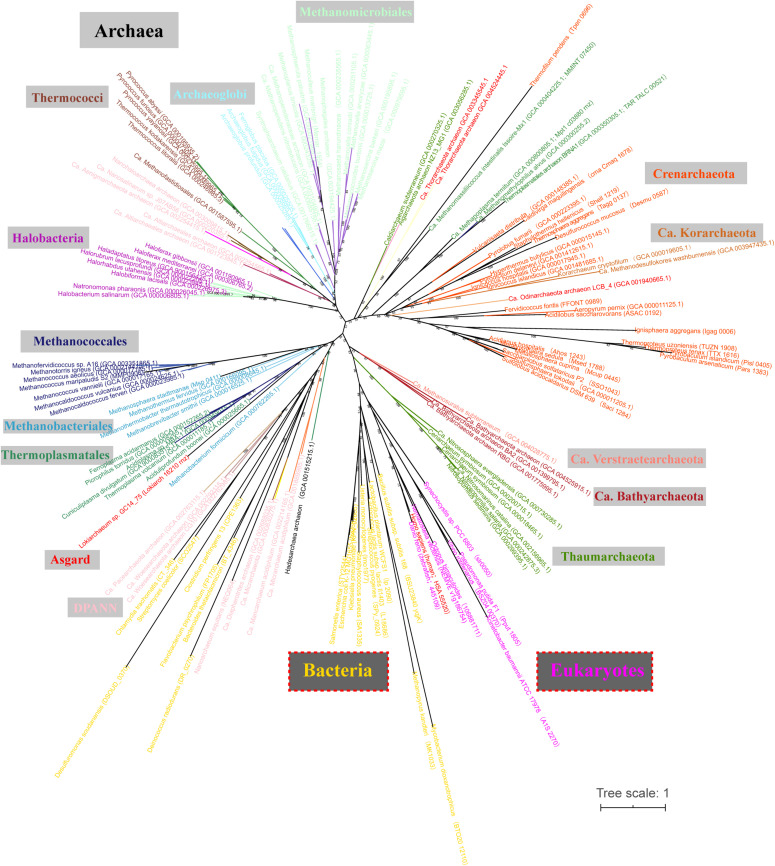
Phylogenetic analysis of the RNase Z orthologs in the representative Archaea, Eukaryotes, and Bacteria. A maximum likelihood phylogenetic tree was constructed based on the protein sequences of RNase Z orthologs retrieved from the KEGG and NCBI database through Blast-P. The tree was constructed based on a maximum likelihood (ML) analysis [IQ-TREE 1.6.12 in the LG + C20 + R4 + F model, 1,000 ultrafast bootstraps replicates (Nguyen et al., 2015; Kalyaanamoorthy et al., 2017; Hoang et al., 2018)], and visualized using iTOL v3 (Letunic and Bork, 2016). Branch support values are indicated by numbers. Scale bar indicates number of substitutions per site.

Through a homolog search, RNase Z orthologs were found in most sequenced archaeal and eukaryotic genomes but not found in half of the searched bacterial genomes and particularly poorly represented in the phylum of Proteobacteria (Supplementary Dataset S1). Phylogenetically, most RNase Z orthologs were congruently clustered as the phylogenetic clades of archaeal species (Figure 1), implying that they could be vertically inherited in Archaea. Noticeably, RNase Z orthologs were found in all searched methanomicrobial archaeal genomes, including the 7th order (Supplementary Dataset S1 and Figure 1), indicating that this protein could be essential to the methanomicrobial archaea. Therefore, two methanomicrobial aRNase Zs, mpy-RNase Z from *M. psychrophilus* that affiliates with the order of *Methanosarcinales* and mmp-RNase Z from *M. maripaludis* that belongs to *Methanococcales*, were chosen for investigation. The two proteins shared 38% amino-acid sequence identity (Supplementary Figure S1), and they were overexpressed in *E. coli* and purified as homogeneous proteins (Supplementary Figure S2). For comparison, a bRNase Z from *B. subtilis*, bsu-RNase Z, was purified and studied in parallel.

### Co^2+^ Enhances the 3′-End Processing Activity of Both the Archaeal and Bacterial RNase Zs

Archaeal RNase (aRNase) Zs affiliate with the β-lactamase family; therefore, the metal ions in facilitating the 3′-end processing activity were first examined. The *in vitro* transcribed bacterial pre-tRNA^bsu–trnI^ and archaeal pre-tRNA^mmp–Arg1^ that carry an 83 nt and a 50 nt 3′-trailer, respectively, (Supplementary Figures S3A,B) were used as substrates. The recombinant mpy-RNase Z and mmp-RNase Z were incubated with each of the synthetic pre-tRNAs in a mixture supplemented with 1 mM of Co^2+^, or Mg^2+^ or Zn^2+^ or Cu^2+^. The bacterial bsu-RNase Z was assayed in parallel. Enzymatic assay determined that in Co^2+^ supplemented reactions, the three RNase Zs invariably cleaved 50–90% of the synthetic pre-tRNAs to generate abundant mature tRNAs (Supplementary Figure S3), while in Mg^2+^ amended reactions generated lower amounts of mature tRNA. These indicated that Co^2+^ enhances the 3′-end processing activity of the three RNase Zs, while Mg^2+^ has a weaker effect. Neither Zn^2+^ nor Cu^2+^ enhanced the activities of the three RNase Zs, and even smear products occurred in the Zn^2+^-supplemented reactions (Supplementary Figure S3), which could be presumably due to Zn^2+^ caused protein precipitation.

**FIGURE 2:**
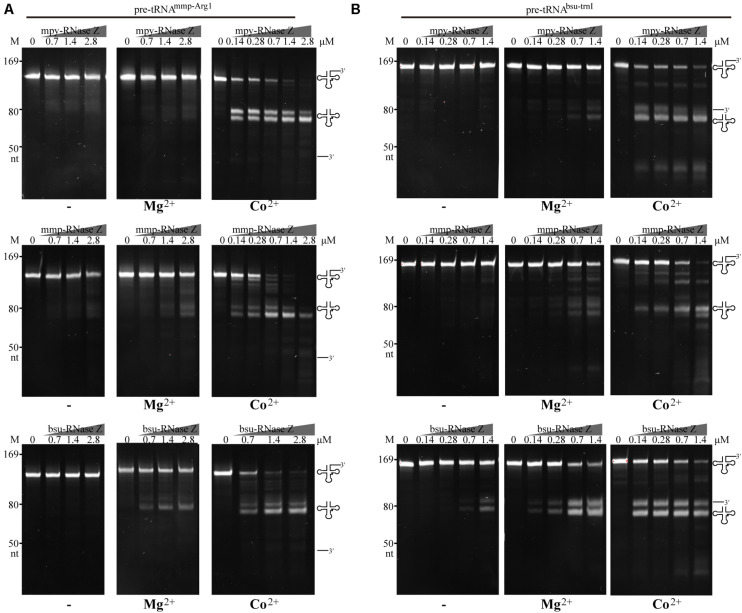
Processing activities of the three RNase Zs on **(A)** the CCA-less pre-tRNA^mmp–Arg1^ and **(B)** pre-tRNA^bsu–trnI^ in the absence (–) or presence of Mg^2+^ or Co^2+^. Pre-tRNA (1.4 pmol) was incubated with mpy-RNase Z, mmp-RNase Z, and bsu-RNase Z at the gradient protein concentrations in a 10 μl reaction for 30 min as described in the “Materials and Methods” section. The cleavage products were analyzed on a 10% polyacrylamide gel with 8 M urea. Migration of the ssRNA markers with indicated size and migration of the pre-tRNAs, mature tRNAs, and 3′-trailer products are indicated at the left and the right of gels, respectively.

Stimulations of Co^2+^ and Mg^2+^ on RNase Zs’ activities were then evaluated on processing the pre-tRNA^mmp–Arg1^ (Figure 2A) and pre-tRNA^bsu–trnI^ (Figure 2B) at gradient protein to RNA ratios of 20:1, 5:1, and 1:1. Enzymatic assays determined that when Co^2+^ amended, at as low as 1:1 of protein to RNA, mature tRNAs generated by the two aRNase Zs were increased for ∼30-fold, while when Mg^2+^ supplemented, mature tRNAs were increased for only ∼two-fold than the reactions without metal-ion (Figures 2A,B, Co^2+^, Mg^2+^ and −). While Co^2+^ showed similar enhancement on bsu-RNase Z, but Mg^2+^ showed stronger stimulation for 5–10-fold more mature tRNAs generated in the Mg^2+^-amended reactions of bsu-RNase Z.

**FIGURE 3:**
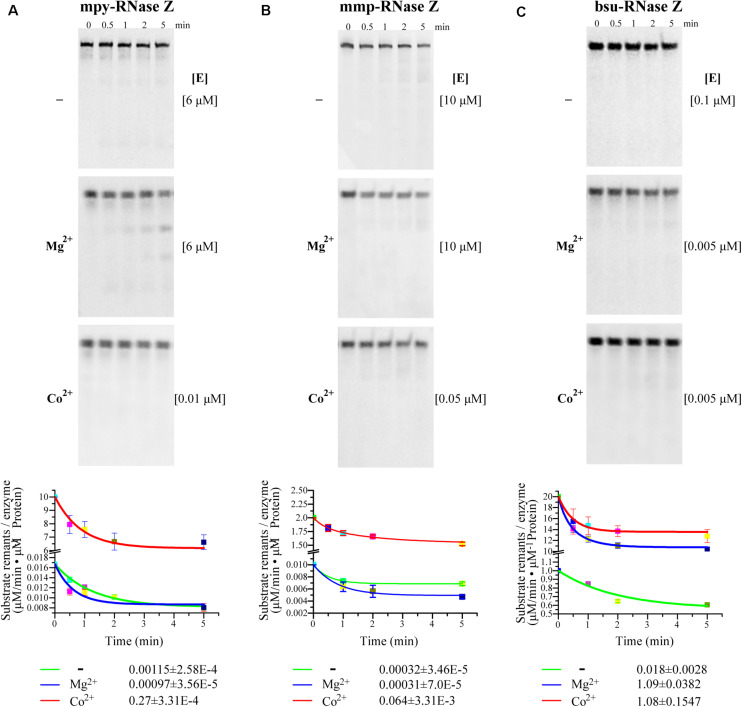
**(A–C)** Initial cleaving velocities (V_0_) of the three RNase Zs on CCA-less pre-tRNAs in the absence (–) or presence of Mg^2+^ and Co^2+^. Through quantification of the residual pre-tRNAs (listed in Table 1) on PAGE gels at the indicated sampling time (upper panels), the initial reaction velocities V_0_ (μM/min residual pre-tRNAsμM^–1^ protein) were calculated based on the pre-tRNA degradation curves representing the first order reactions. [E], enzyme concentrations used in the reactions of each panel. The mean ± s.d from three experimental replicates are shown.

To quantify the effects of Co^2+^ and Mg^2+^, the initial cleavage velocities (V_0_) of the three RNase Zs were determined using CCA-less pre-tRNAs as substrates. Through quantifying the substrate residuals within the linear phase during the initial 5 min, it determined that the V_0_ (μM/min^–1^ pre-tRNA⋅μM^–1^ protein) of mpy-RNase Z and mmp-RNase Z were increased for 235- and 200-fold, respectively, by Co^2+^ supplementation but were not obviously increased by Mg^2+^ supplementation (Figures 3A,B). Whereas, the V_0_ of bsu-RNase Z was equally enhanced by Co^2+^ and Mg^2+^ with ∼60-fold increase than that of no ion supplementation (Figure 3C).

Further, through replicative quantifying the cleaving velocities on a range of (0.025–1 μM) of the CCA-less pre-tRNA concentrations, kinetic parameters K_m_ and k_cat_ were further compared for the three RNase Zs in reactions with or without Co^2+^ (Table 1 and Supplementary Figure S4). Supplementation of Co^2+^ slightly (0. 43-, 0. 47-, and one-fold) affected the K_m_ values but dramatically elevated the k_cat_ values of mpy-RNase Z, mmp-RNas Z, and bsu-RNase Z for 607-, 1400-, and 34.4-fold, respectively, and accordingly significantly elevated the cleavage efficiencies (k_cat_/K_m_) of the three RNase Zs for 1400-, 2990-, and 34-fold, respectively (Table 1 and Supplementary Figure S4). Noteworthily, bsu-RNase Z exhibited higher cleavage efficiency than the two aRNase Zs. The k_cat_/K_m_ value of bsu-RNase Z is 158- and 644-fold higher than that of mpy- and mmp-RNase Z, respectively, in no metal-ion reactions (Table 1), and the cleavage velocities of the three RNase Zs were evaluated ordered as bsu-RNase Z, mpy-RNase Z, mmp-RNase Z (high to low) (Figure 3). Collectively, Co^2+^ markedly stimulates the activities of the three RNase Zs, and Mg^2+^ also enhances the activity of bsu-RNase Z.

### The aRNase Zs Indiscriminately Process Pre-tRNAs With or Without a CCA Motif

**FIGURE 4:**
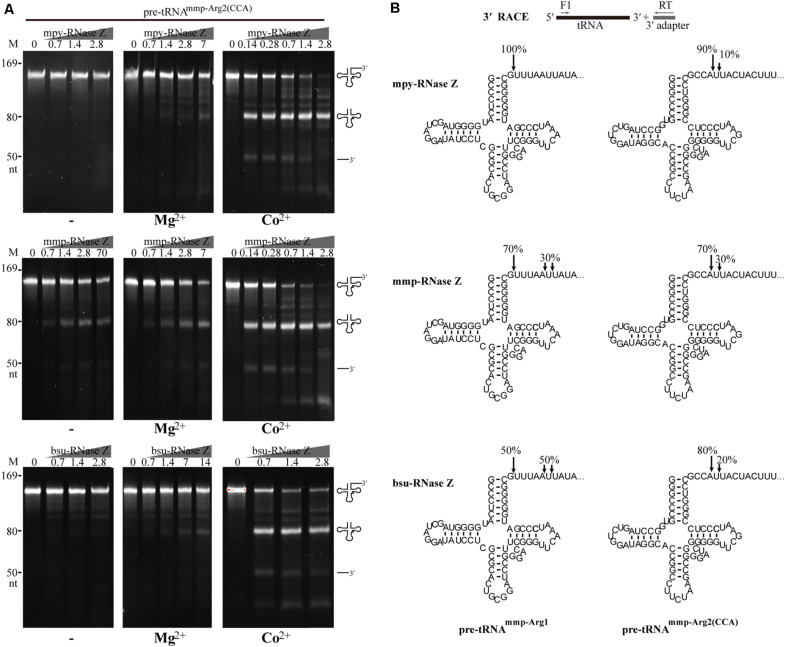
Processing activities of the three RNase Zs on an archaeal CCA-containing pre-tRNA^mmp–Arg2(CCA)^. **(A)** Nuclease assays were performed in the absence (–) or presence of Mg^2+^ or Co^2+^. Pre-tRNA (1.4 pmol) was incubated with RNase Z at gradient concentrations in a 10 μl reaction mixture as described in the “Materials and Methods” section. Cleavage products were analyzed on a 10% polyacrylamide gel with 8 M urea. Migrations of ssRNA markers and the pre-tRNAs, mature tRNAs, and 3′-trailer products are shown at the left and the right of the gels, respectively. **(B)** 3′-RACE identifying the cleavage sites of the three RNase Zs on CCA-less pre-tRNA^mmp–Arg1^ and CCA-containing pre-tRNA^mmp–Arg2(CCA)^. The cleavage percentages at each site are indicated. The schematic diagram at top briefly illustrates the 3′-RACE workflow. The horizontal gray arrows show the primers used for reverse transcription and PCR amplification.

Distinct from the eukaryotic tRNA genes, varying proportions of the prokaryotic tRNA genes genetically encode a CCA motif. For example, 13 of the 52 tRNA genes (25%) in *M. psychrophilus* R15 and 10 of the 38 (26%) in *M. maripaludis* S2 encode the CCA motif downstream the discriminator nucleotide, respectively (Supplementary Datasets S2, S3). To evaluate the activity of aRNase Zs in processing pre-tRNAs with the encoded CCA motif, a CCA-containing pre-tRNA^mmp–Arg2(CCA)^ was used as substrate and bsu-RNase Z was included in parallel (Figure 4A). Similar to the activity on CCA-less pre-tRNAs (Figure 2), the three RNase Zs all exhibited Co^2+^ and Mg^2+^ stimulated activities on CCA-containing pre-tRNA^mmp–Arg2(CCA)^; namely, when Co^2+^ supplemented, they efficiently cleaved the CCA-containing pre-tRNA at equivalent low protein to RNA ratios of 1:1 and 5:1 as that on CCA-less ones (Figure 4A). Interestingly, mmp-RNase Z even exhibited a higher cleavage activity on CCA-containing pre-tRNAs in the reactions without metal-ion or with Mg^2+^ (Figures 2, 4A). While the CCA motif appeared to suppress the bsu-RNase Z’s activity in Mg^2+^ supplemented reaction, as less cleavage products were generated from the CCA-containing pre-tRNA^mmp–Arg2(CCA)^ than from the CCA-less pre-tRNAs (Figures 2, 4A).

Next, 3′-RACE was performed to determine the cleavage sites in CCA-containing pre-tRNA^mmp–Arg2(CCA)^ and CCA-less pre-tRNA^mmp–Arg1^ of the three RNase Zs in the presence of Co^2+^ (Figure 4B). The primary cleaved mature tRNA products were recovered for 3′-end sequencing, which showed that the CCA-less pre-tRNA^mmp–Arg1^ was cleaved primarily downstream the discriminator nucleotide, representing 100%, 70%, and 50% of the cleaving sites generated by mpy-RNase Z, mmp-RNase Z, and bsu-RNase Z, respectively. The remaining 30% and 50% cleavage sites of mmp-RNase Z and bsu-RNase Z were located five or six nucleotides downstream the discriminator nucleotide, respectively (Figure 4B). Unexpectedly, the CCA-containing pre-tRNA^mmp–Arg2(CCA)^ was mainly cleaved immediate downstream the CCA motif, representing 90%, 70%, and 80% cleaving sites, respectively. The remaining cleavage sites (10%, 30%, and 20%) all located just one nucleotide downstream the CCA motif. Consequently, cleavages of the three RNase Zs on CCA-less pre-tRNAs result in mature tRNA that carries the 3′-end discriminator nucleotide, while cleavages on CCA-containing pre-tRNAs generate mature tRNA ended with the CCA triplet.

**FIGURE 5:**
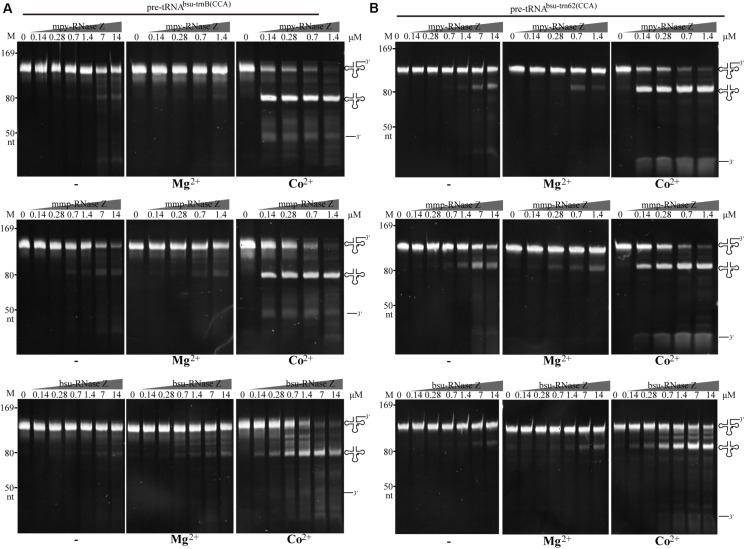
Processing of the bacterial CCA-containing pre-tRNA^bsu–trnB(CCA)^ **(A)** and pre-tRNA^bsu–trn62(CCA)^ **(B)** by the three RNase Zs in the absence (–) or presence of Mg^2+^ or Co^2+^. Pre-tRNA (1.4 pmol) was incubated with RNase Zs at the indicated concentrations in a 10 μl reaction mixture as described in the “Materials and Methods” section. Cleavage products were separated on a 10% polyacrylamide gel with 8 M urea. Migrations of ssRNA markers and the pre-tRNAs, mature tRNAs, and 3′ trailer products are shown at the left and at the right of the gels, respectively.

Further, three more CCA-containing pre-tRNAs, the *B. subtilis* pre-tRNA^bsu–trnB(CCA)^ and pre-tRNA^bsu–t62(CCA)^ (Figure 5), and the *M. psychrophilus* pre-tRNA^mpy–Arg2(CCA)^ (Supplementary Figure S5), were used as substrates to evaluate the cleavage specificity of the three prokaryotic RNase Zs. Similar to the results with pre-tRNA^mmp–Arg2(CCA)^ (Figure 4A), the two aRNase Zs at a protein to RNA ratio of 1:1 produced significant amounts of mature tRNAs from the three CCA-containing pre-tRNAs when Co^2+^ supplemented (Figures 5A,B and Supplementary Figure S5), further confirming that they process CCA-containing pre-tRNAs with a comparable activity as CCA-less ones (compare with Figure 2). The two aRNase Zs even exhibited higher activity on CCA-containing than on CCA-less pre-tRNAs in the absence of metal-ion or with Mg^2+^-supplementation (compare Figures 2, 5, and Supplementary Figure S5). However, bsu-RNase Z produced less cleaved products from CCA-containing pre-tRNAs than the two aRNase Zs (Figure 5 and Supplementary Figure S5), indicating a suppression of the CCA motif on the activity of bRNase Z. The CCA motif inhibition on bsu-RNase Z was even more significant in Mg^2+^-supplemented condition, as a 50–100-fold higher protein concentration was required to produce comparable amounts of cleaved product from CCA-containing as CCA-less pre-tRNAs (comparing Figures 2, 5 and Supplementary Figure S5).

The kinetic parameters, K_m_ and k_cat_, of the three RNase Zs were then assayed to quantify the cleavage efficiencies on CCA-less vs. CCA-containing pre-tRNAs (Table 1). In Co^2+^-amended reactions, K_m_ values of mpy-RNase Z, mmp-RNase Z, and bsu-RNase Z were 1. 65-, 0. 69-, and 0.63-fold changed, respectively, on CCA-containing vs. CCA-less pre-tRNAs, suggesting that the pre-tRNA binding affinities of them were slightly affected by the CCA-motif. While, the k_cat_ values of mpy-RNase Z and mmp-RNas Z on CCA-containing pre-tRNAs were 1.38- and 2.14-fold, and the k_cat_/K_m_ were 0.83- and 3-fold than those on CCA-less pre-tRNAs, respectively. In contrast, the k_cat_ and k_cat_/K_m_ values of bsu-RNase Z on CCA-containing pre-tRNA were 18.7-fold and 11.9-fold lower than those on CCA-less one, respectively. These results indicated that in the presence of Co^2+^, the two aRNase Zs retain nearly indiscriminate cleavage efficiencies on CCA-containing and CCA-less pre-tRNAs, while the catalysis efficiency of the bacterial bsu-RNase Z is inhibited by the CCA-motif. Consistently, the initial cleavage velocities of the three RNase Zs also supported these conclusions (Supplementary Figure S6A).

Based on that 100-fold higher bsu-RNase Z protein was required to process the CCA-containing than the CCA-less pre-tRNA in Mg^2+^-amended reaction, the CCA-motif was reported to inhibit the activity of bsu-RNase Z (Pellegrini et al., 2003). In this study, the initial velocity of bsu-RNase Z on CCA-containing pre-tRNA was determined to be 1520-fold lower than that on CCA-less one in Mg^2+^-amended reaction (Supplementary Figure S6B), while be only 5.8-fold lower in Co^2+^ supplementation (Supplementary Figure S6A). Therefore, these indicated that although the encoded CCA motif severely inhibits the activity of bsu-RNase Z, Co^2+^-supplementation could dramatically ameliorate the inhibition.

### The aRNase Zs Require 5′ Matured Pre-tRNA for 3′-End Processing

**FIGURE 6:**
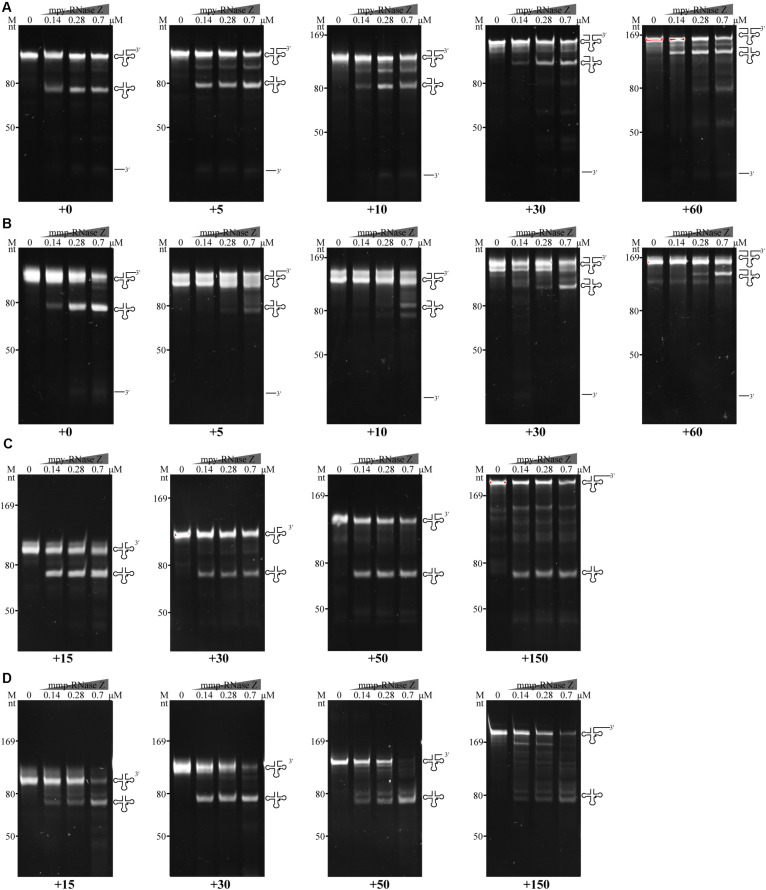
Effects of 5′ and 3′ extensions on the 3′-end processing of pre-tRNAs by **(A,C)** mpy-RNase Z and **(B,D)** mmp-RNase Z. **(A,B)** Pre-tRNA^mpy–Arg1^ and pre-tRNA^mmp–Arg1^ carrying a 30 nt 3′-trailer and 5′ extensions with 0, 5, 10, 30, or 60 nt were used as substrates. **(C,D)** Pre-tRNA^mpy–Arg1^ and pre-tRNA^mmp–Arg1^ with no 5′ extension but carrying 3′-trailers of 15, 30, 50, or 150 nt were used as substrates. Pre-tRNA (1.4 pmol) was incubated with purified RNase Zs at gradient concentrations in a 10 μl reaction mixture as described in the “Materials and Methods” section. The cleavage products were separated on a 10% polyacrylamide gel with 8 M urea. Migration of ssRNA markers, the pre-tRNAs, mature tRNAs, and 3′-trailer products are indicated at the left and at the right of the gels, respectively.

Most of RNase Zs analyzed so far appear requiring a mature tRNA 5′-end for 3′-end processing (Kunzmann et al., 1998; Nashimoto et al., 1999b; Schierling et al., 2002; Pellegrini et al., 2003), so the 5′-leader removal is assumed preceding the 3′-end processing of pre-tRNAs by RNase Z (Redko et al., 2007). To evaluate the effect of the pre-tRNA 5′ extensions on the 3′-end processing of aRNase Zs, pre-tRNA^mpy–Arg1^ and pre-tRNA^mmp–Arg1^ that carry varying lengths of 5′ extensions were used as substrates. The cleavage assays showed that although the two aRNase Zs processed the 5′ extended pre-tRNAs, lower 3′-trailer processing activities were observed for those with longer 5′ extensions. In detail, ≥10 nt 5′ extensions markedly suppressed the 3′-end processing activity of mpy-RNase Z (Figure 6A), while a 5 nt-5′ extension already inhibited mmp-RNase Z (Figure 6B).

Effect of the 3′-trailer lengths on the activities of two aRNase Zs were also assayed. The results showed that mpy-RNase Z and mmp-RNase Z could efficiently cleave all tested pre-tRNAs with various lengths of 3′-trailer, although reduced activity was found on a 150 nt-3′-trailer (Figures 6C,D). These results demonstrate that the two aRNase Zs only efficiently process the 3′-ends of pre-tRNAs that have matured 5′-ends, but regardless, the 3′-trailer lengths, that is, RNase P cleavage to produce a mature 5′-end should precede the 3′-trailer processing by RNase Z.

### The aRNase Zs but Not the bRNase Z Process Intron-Containing Pre-tRNAs

**FIGURE 7:**
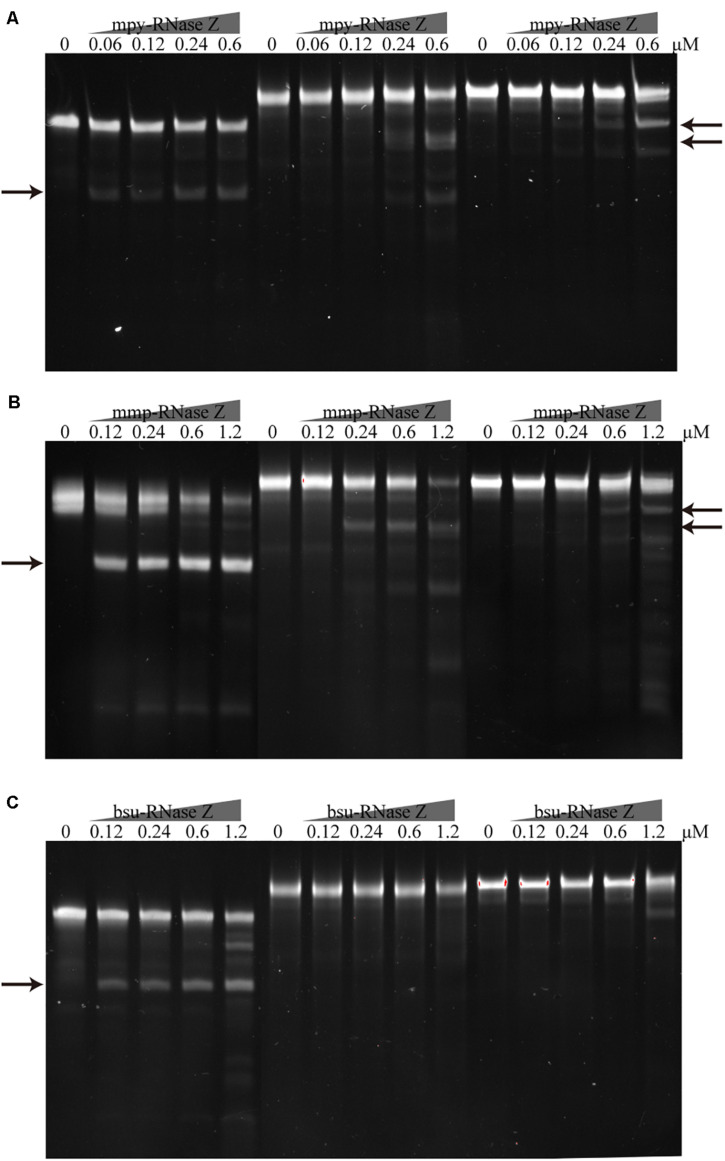
Processing of the intron containing pre-tRNAs by the archaeal and bacterial RNase Zs. The intron-less pre-tRNA^mpy–Arg1^ and the intron-containing pre-tRNA^mpy–Arg3(intron)^ and pre-tRNA^mpy–Tyr(intron)^ were each incubated with **(A)** mpy-RNase Z, **(B)** mmp-RNase Z, and **(C)** bsu-RNase Z. Pre-tRNA^mpy–Arg3(intron)^ and pre-tRNA^mpy–Tyr(intron)^ contain 24 and 38 nt introns, respectively. Pre-tRNA (1.2 pmol) was incubated with purified RNase Z at the indicated concentrations in a 10 μl reaction mixture as described in the “Materials and Methods” section. The cleavage products were separated on a 10% polyacrylamide 8 M urea gel and are indicated by arrows.

Considering that some archaeal pre-tRNAs contain introns (Tocchini-Valentini et al., 2005), we then evaluated the processing activities of the two aRNase Zs on pre-tRNA^mpy–Arg3(intron)^ and pre-tRNA^mpy–Tyr(intron)^ that contain 24 nt- and 38 nt-long introns, respectively. Cleavage assays determined that the two aRNase Zs were capable of cleaving the intron-containing pre-tRNAs but with lower efficiency than on intron-less pre-tRNAs. In contrast, no detectable activity was found for bsu-RNase Z on processing the intron-containing pre-tRNAs (Figure 7). The capability of the aRNase Zs, but not the bRNase Z, in processing the intron-containing pre-tRNAs complies with the fact that the intron-carrying pre-tRNAs are present in archaea but not in bacteria (Tocchini-Valentini et al., 2005).

### Two aRNase Zs Process Aberrant Pre-tRNAs, but the Acceptor Stem Is Indispensable

**FIGURE 8:**
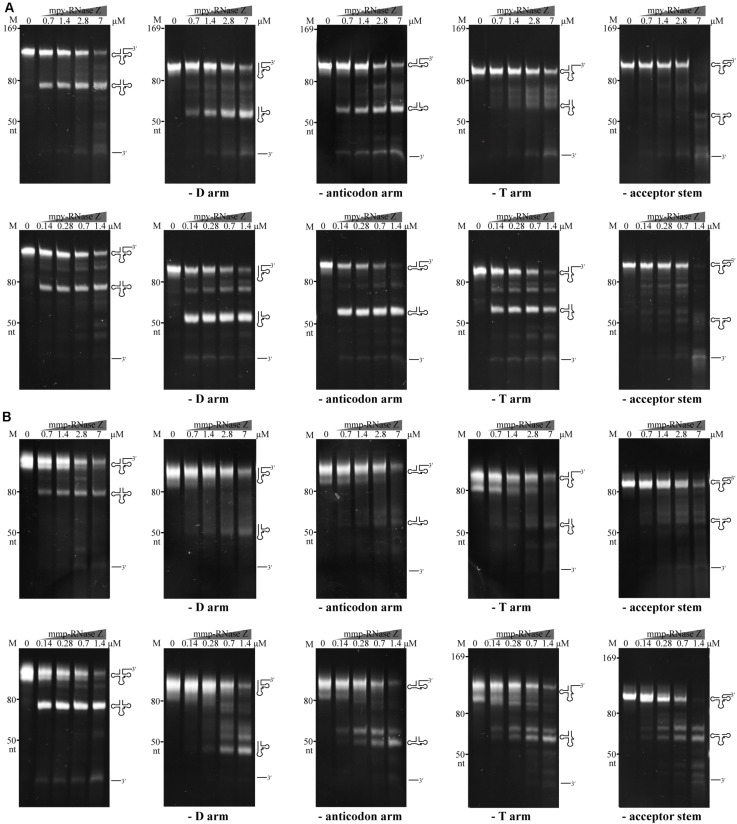
The tRNA element requirements of mpy-RNase Z **(A)** and mmp-RNase Z **(B)** for the pre-tRNA 3′-end processing. The pre-tRNA mutants that have mature 5′-end but lacking the D arm, the anticodon arm, the T arm, or the acceptor stem were synthesized. Each pre-tRNA variant (1.4 pmol) was incubated with the purified RNase Zs at indicated concentrations in a 10 μl reaction mixture as described in the “Materials and Methods” section. The cleavage products were separated on a 10% polyacrylamide 8 M urea gel. Migration of ssRNA markers, and that of the pre-tRNA variants, mature tRNAs, and 3′-trailer products are indicated at the left and at the right of the gels, respectively.

Next, we assayed the requirements of tRNA elements by aRNase Zs in processing the pre-tRNA 3′-end. An array of tRNA variants that lack the D arm, anticodon arm, T arm, or acceptor stem but carry a 30 nt-3′-trailer were used as substrates (Figure 8). Each of the aberrant pre-tRNAs was incubated with the two aRNase Zs. In the presence of Co^2+^, the two aRNase Zs processed all the pre-tRNA variants at a comparable efficiency as the wild-type pre-tRNA, except for the variant that lacks the acceptor stem. However, in Mg^2+^-supplemented reactions, lacking any element resulted in a markedly reduced processing efficiency of mmp-RNase Z; while deletion of the T arm or acceptor stem, but not the D and anticodon arms, suppressed the activity of mpy-RNase Z. This indicates that as long as Co^2+^ is present, the aRNase Zs are capable of processing pre-tRNAs without the D, the anticodon, or the T arm, but the acceptor stem is indispensable, implying that the aRNase Zs could have a broad *in vivo* substrate spectrum in addition to pre-tRNAs.

## Discussion

Thus far, our knowledge about the properties of methanomicrobial aRNase Zs, particularly in processing the CCA-containing pre-tRNAs, remains limited. The present study has comprehensively examined the biochemical characteristics of two aRNase Zs from methanomicrobial archaea. We found that Co^2+^ markedly activates the pre-tRNA 3′-end processing efficiencies of the two aRNase Zs for 1440- and 2990-fold, respectively, and even is indispensable to the aRNase Zs. Distinctively, the two aRNase Zs indiscriminately process CCA-containing and CCA-less pre-tRNAs with similar catalytic efficiency (k_cat_/K_m_) and generate the mature tRNA ended with CCA in the former and the discriminator nucleotide in the latter, respectively. Moreover, Co^2+^ not only activates the pre-tRNA processing activity of the bRNase Z but also ameliorates the CCA motif inhibitory effect from 1520-fold to 5.8-fold. Noticeably, the two methanomicrobial aRNase Zs are capable of processing intron-containing pre-tRNAs and aberrant pre-tRNA mutants that lack the T, D, or anticodon arm, but require the acceptor stem and mature 5′-end for 3′-end processing. Collectively, this work elucidates the characteristics of methanomicrobial aRNase Zs, in particular the capability of processing CCA-containing pre-tRNAs, which could be shared by aRNase Z orthologs that are ubiquitously distributed in archaea.

### Co^2+^, Mn^2+^, and Mg^2+^ Appear to Be Required by Most Prokaryotic RNase Zs

Though RNase Z affiliates with the β-lactamase family metalloproteins and Zn^2+^ (one or two) coordinated in the metallo-β-lactamase domain has been observed in the RNase Zs from *B. subtilis* (Li de la Sierra-Gallay et al., 2005), T. *maritima* (Ishii et al., 2005), and *E. coli* (Kostelecky et al., 2006), Zn^2+^ addition neither promotes the activities of the archaeal nor the bRNase Zs (Supplementary Figure S3). On the contrary, this work found that Co^2+^ supplementation markedly enhances the 3′-end processing activities of both the archaeal and bRNase Zs, regardless of the pre-tRNAs from archaeal or bacteria or containing the CCA motif or not (Table 1 and Figures 2, 3, 4A, 5 and Supplementary Figures S3–S6). In addition to Co^2+^, Mg^2+^ also promotes the activity of the bacterial bsu-RNase Z (Figures 2, 3, 5). Co^2+^ stimulation on *E. coli* RNase Z was reported in an earlier study (Asha et al., 1983), and Mn^2+^ has similar effect in stimulating the activities of RNase Zs from *T. maritima* (Minagawa et al., 2006), *A. thaliana* (Spath et al., 2007), *H. volcanii*, and *P. furiosus* (Spaeth et al., 2008). Therefore, the stimulatory effects of Co^2+^, Mn^2+^, and Mg^2+^ could be the common property among RNase Zs from eukaryotes, archaea, and bacteria. These metal ions might help pre-tRNA fold properly, or induce RNase Z to attain an active conformation and/or assist RNase Z to interact with pre-tRNA correctly. However, to unveil the underlying mechanisms, further studies are required, such as to solve the structures of the apo or pre-tRNA-complexed RNase Z with or without a metal ligand to compare the detailed conformation changes.

Although bsu-RNase Z and the two aRNase Zs have similar key elements and conserved sequences as indicated by the protein sequence alignment (Supplementary Figure S1), higher cleavage velocity and efficiency have been found for bsu-RNase Z even without the addition of metal ions (Figure 3 and Table 1). This could be attributed to a higher metal affinity of bsu-RNase Z, in which more metal ions have been already sequestered during purification. Supportively, a higher EDTA concentration was needed to inhibit the cleavage activity of bsu-RNase Z than that to the two aRNase Zs (Supplementary Figure S7). Moreover, the aRNase Zs rely more on Co^2+^ than bsu-RNase Z (Supplementary Figure S3 and Figures 2, 3 and Table 1), while the latter is also activated by Mg^2+^ (Figures 2, 3 and Table 1), implying that the aRNase Zs have a better adaptation to Co^2+^ and bsu-RNase Z to Mg^2+^. It is assumed that bacteria have higher cellular levels of Mg^2+^ and Mn^2+^, but the methanomicrobial archaea contain higher Co^2+^. Cobalt is used as a metal ligand in some methanomicrobial enzymes, for example, the methanol and methyl amine methyltransferases are all corrinoid proteins, and Co^2+^ is routinely supplemented in the culture media of methanogens (Sarmiento et al., 2011). This could be a circumstantial evidence that aRNase Zs rely more on Co^2+^, while the bRNase Zs are better adapted to Mg^2+^.

### Co^2+^ Is Specifically Required for RNase Zs in Processing CCA-Containing Pre-tRNAs

This work found that the aRNase Zs exhibit a comparable activity of processing the CCA-containing and CCA-less pre-tRNAs (Figures 2–5 and Supplementary Figures S4–S6) and retain similar k_cat_/K_m_ values (Table 1) and generate matured tRNA 3′-ends with CCA in the former and the discriminator nucleotide in the latter. Therefore, aRNase Z could be the single ribonuclease functioning in the maturation of tRNA 3′-ends in archaea; this is different from that in bacteria, in which not only RNase Z-dependent endonucleolytic maturation but also additional exonucleolytic pathway through collaboration of several enzymes both exist (Redko et al., 2007). The CCA-containing pre-tRNA genes, with varying proportions, are distributed in many archaeal genomes; for example, 26% and 25% of tRNA genes in *M. maripaludis* S2 and *M. psychrophilus* R15 contain the CCA motif (Supplementary Datasets S2, S3), respectively. Thus, we hypothesized that RNase Z-mediated single-step endoribonucleolytic cleavage plays a primary role in archaeal tRNA 3′-end maturation, as that in eukaryotes (Castano et al., 1985; Frendewey et al., 1985; Stange and Beier, 1987; Oommen et al., 1992). Although this hypothesis is not yet verified *in vivo*, the gene-encoding mmp-RNase Z has been determined as essential in *M. maripaludis* (Sarmiento et al., 2013), providing a circumstantial evidence for the key functions of aRNase Z, presumably through the single-step tRNA 3′-end maturation.

Consistent with the previous findings (Pellegrini et al., 2003), the present work found that the CCA motif exerts an obvious inhibitory effect on the bacterial bsu-RNase Z. However, this inhibitory effect was significantly reduced when Co^2+^ was amended (Figures 3–5 and Supplementary Figures S5, S6 and Table 1). Similarly, the *E. coli* RNase Z was reported to process the CCA-containing pre-tRNAs in the presence of Co^2+^ (Dutta et al., 2012), and actually involved in the maturation of all 86 CCA-containing pre-tRNAs when RNases T, PH, D, and II are absent (Kelly and Deutscher, 1992), so it can be one primary player in tRNA 3′-end maturation as well. The *T. maritima* RNase Z also exhibited a cleavage activity downstream the CCA motifs of 45 CCA-containing tRNAs (Minagawa et al., 2004). Thus, both bacterial and aRNase Zs are capable of processing CCA-containing pre-tRNAs and expose the genetically encoded CCA triplet. This also suggests that RNase Z-mediated one-step processing on the CCA-containing pre-tRNAs could be a widely distributed mode in prokaryotes.

### Substrate Recognition and Processing Order of the Methanomicrobial aRNase Zs in Pre-tRNA Maturation

The present work found that ≥10 nt 5′ extensions markedly suppressed the pre-tRNAs processing efficiency of the aRNase Zs, suggesting that a mature 5′-end is a precondition for 3′ maturation of pre-tRNAs (Figures 6A,B). Similar observations were found in *B. subtilis* RNase Z, which exhibited a reduced 3′-end processing activity on pre-tRNAs having ≥33 nt 5′ extensions (Pellegrini et al., 2003); while the pig liver RNase Z even lost its 3′-end processing activity on pre-tRNAs with 5′ extensions >9 nt (Nashimoto et al., 1999a). Thus, analogous to the eukaryotic and bacterial pre-tRNA processing procedures, RNase P-mediated 5′-end processing could precede the 3′-end processing by RNase Z in methanomicrobial archaea.

While, the 3′ extension lengths did not exhibit inhibitory effects on the two aRNase Zs, which showed nearly indiscriminate processing activities on pre-tRNAs with diverse 3′ extensions (Figures 6C,D). Moreover, the two aRNase Zs are capable of processing intron-containing pre-tRNAs though with a lower efficiency (Figure 7), so they could be involved in maturation of the inherited intron-containing pre-tRNAs in archaea (Tocchini-Valentini et al., 2005; Supplementary Dataset S2). This also suggests that aRNase Zs could process the 3′-end before the intron removal when the tRNA precursors are at high concentrations, while intron scissoring may occur first when the precursors are at physiological concentrations.

In addition, the two methanomicrobial aRNase Zs indiscriminately process the homologous and heterologous tRNA precursors, indicating that they recognize tRNA structures but not the sequences. Enzymatic assays on the pre-tRNA element mutants determined that the acceptor stem, but not the D, anticodon, and T arms, is required for aRNase Zs in the tRNA 3′-end maturation in the presence of Co^2+^ (Figure 8). In support of these observations, a tRNA-bound structure of the *B. subtilis* RNase Z has revealed the direct interactions of the T-arm and the acceptor stem with the flexible arm and helix α7 of the RNase Z dimer, while the D-arm and anticodon loop are dispensable in the interaction, and the tRNA phosphodiester backbone is primarily recognized (Li de la Sierra-Gallay et al., 2006). Therefore, these interactions enable RNase Zs adapted to a wide variety of tRNA substrates.

In conclusion, in the presence of Co^2+^, the methanomicrobial aRNase Zs are capable of processing the 3′-end of various pre-tRNA species, including CCA-containing and CCA-less, intron-containing, and T-, D-, and anticodon-arm-lacking tRNA precursors, hinting their pivotal roles in pre-tRNA 3′-end maturation and a potential broad substrate spectrum, so aRNase Zs could fulfill a plethora of functions in RNA metabolism in archaea.

## Data Availability Statement

All datasets generated for this study are included in the article/Supplementary Material.

## Author Contributions

JL and XD conceptualized the experiments and acquired funding. XW, XG, and JL designed and performed the biochemical experiments. LY performed the protein expression and 3′-RACE experiments. All of the researchers interpreted the experimental data and assisted with the preparation of the manuscript. JL, DL, and XD wrote the manuscript. All authors approved the final manuscript.

## Conflict of Interest

The authors declare that the research was conducted in the absence of any commercial or financial relationships that could be construed as a potential conflict of interest.

## Supplementary Material

FIGURE S1 Sequence alignment of the RNase Z family proteins. The protein sequences were aligned using ClustalW program (Larkin et al., 2007) and the diagram was prepared using ESPript program (Gouet et al., 1999). Identical residues are highlighted with white type on a red background and similar residues are shown as red type. *Methanolobus psychrophilus* RNase Z (Mpsy_2804) and *Methanococcus maripaludis* (MMP0906) shares 38% and 58% amino-acid sequence identity and similarity, respectively. Secondary structural elements of bsu-RNase Z (PDB ID: 1Y44) are shown on the top of the sequence. *Bacillus subtilis* RNase Z (bsu: Bsu23480) shares 48%, 42%, 42%, 36%, 36%, 32%, 33%, 33%, and 28% amino acid sequence identity to *Escherichia coli* RNase Z (eco: b2268), *M. maripaludis* RNase Z (mmp: MMP0906), *Methanocaldococcus jannaschii* RNase Z (mja: MJ_1502), *M. psychrophilus* RNase Z (mpy: Mpsy_2804), *Haloferax volcanii* RNase Z (hvo: HVO_0144), *Pyrococcus furiosus* RNase Z (pfu: PF12345), *Thermoplasma acidophilum* RNase Z (tac: Tal155), *Pyrobaculum aerophilum* RNase Z (pai: PF12345), and *Thermotoga maritima* RNase Z (tma: TM0864), respectively.

FIGURE S2 SDS-PAGE of the three RNase Zs tested in this study. The purified His_6_-tagged recombinant RNase Z proteins on 12% SDS-PAGE were shown. M, the protein ladders indicated at the left to identify the migration positions of the proteins.

FIGURE S3 Stimulatory effects of Co^2+^ or Mg^2+^ on the tRNA 3′-end processing activity of the archaeal and bacterial RNase Zs. Two pre-tRNAs were used as substrates, that is, **(A)** *M. maripaludis* S2 pre-tRNA^mmp–Arg1^ and **(B)** *B. subtilis* pre-tRNA^bsu–trnI^. Purified mpy-RNase Z from *M. psychrophilus*, mmp-RNase Z from *M. maripaludis*, and bsu-RNase Z from *B. subtilis* were assayed in the absence (−) or presence (+) of 1 mM Zn^2+^, Cu^2+^, Co^2+^, or Mg^2+^. Pre-tRNA (1.4 pmol) was incubated with 0.7 μM mpy-RNase Z, 0.7 μM mmp-RNase Z, and 0.28 μM bsu-RNase Z at 37°C for 30 min. The cleavage products were separated on a 10% polyacrylamide 8 M urea gel. Migration of the ssRNA markers with indicated lengths and migration of the pre-tRNAs, mature tRNAs, and 3′-trailer products are marked at the left and at the right of gels, respectively.

FIGURE S4 The Lineweaver–Burk plots of the kinetic parameters of the three RNase Zs for processing the CCA-containing (CCA+) and CCA-less (CCA−) pre-tRNAs in the absence (−) or presence (+) of Co^2+^. A range of (0.025–1 μM) pre-tRNA (Table 1) substrate concentrations were assayed for each tested RNase Z at 37°C. The initial velocity (V_0_) of RNase Z at each substrate concentration was determined through quantifying the substrate residuals in the linear phase during the initial 5 min similarly as that shown in Figure 3. The kinetic parameters of K_m_, V_max_, and k_cat_ were obtained by fitting the Lineweaver–Burk plotting data to the Michaelis-Menten equation.

FIGURE S5 Ribonuclease assays of the processing activity of the three RNase Zs on the CCA containing *M. psychrophilus* pre-tRNA^mpy–Arg2(CCA)^ in the absence (−) or presence of Mg^2+^ or Co^2+^. Pre-tRNA (1.4 pmol) was incubated with purified recombinant RNase Z at gradient concentrations in a 10 μl nuclease reaction as described in the “Materials and Methods” section. Cleavage products were separated on a 10% polyacrylamide 8 M urea gel. The migration of ssRNA markers and the migration of pre-tRNAs, mature tRNAs, and 3′-trailer products are labeled at the left and at the right of the gels, respectively.

FIGURE S6 Comparison of the initial velocities of the three RNase Zs for processing the CCA-less and CCA-containing pre-tRNAs in Co^2+^ supplementation **(A)** and in Mg^2+^ supplementation for bsu-RNase Z **(B)**. Initial reaction velocities (V_0_) were determined by quantifying the residual pre-tRNA substrate amounts (listed in Table 1) on PAGE gels at each sampling time, which are shown as attenuation curves of pre-tRNA remnants per the enzyme amounts in the upper panels. Values of V_0_ (μM/min residual pre-tRNAs⋅μM^–1^ protein) indicated below the panels are the mean ± s.d from three experimental replicates.

FIGURE S7 EDTA inhibition on the 3′-processing activity of the three RNase Zs. EDTA concentrations were shown on the top of the gel. CK, control reactions without enzyme.

TABLE S1 Primers used in this study.

TABLE S2 Sequences of RNA substrates used in this study.
