# Supplementary figures and images for: Characterization of the Methanomicrobial Archaeal RNase Zs for Processing the CCA-Containing tRNA Precursors
Source: Front Microbiol. 2020 Aug 25;11:1851. doi: 10.3389/fmicb.2020.01851 (PMC7479834; doi:10.3389/fmicb.2020.01851)

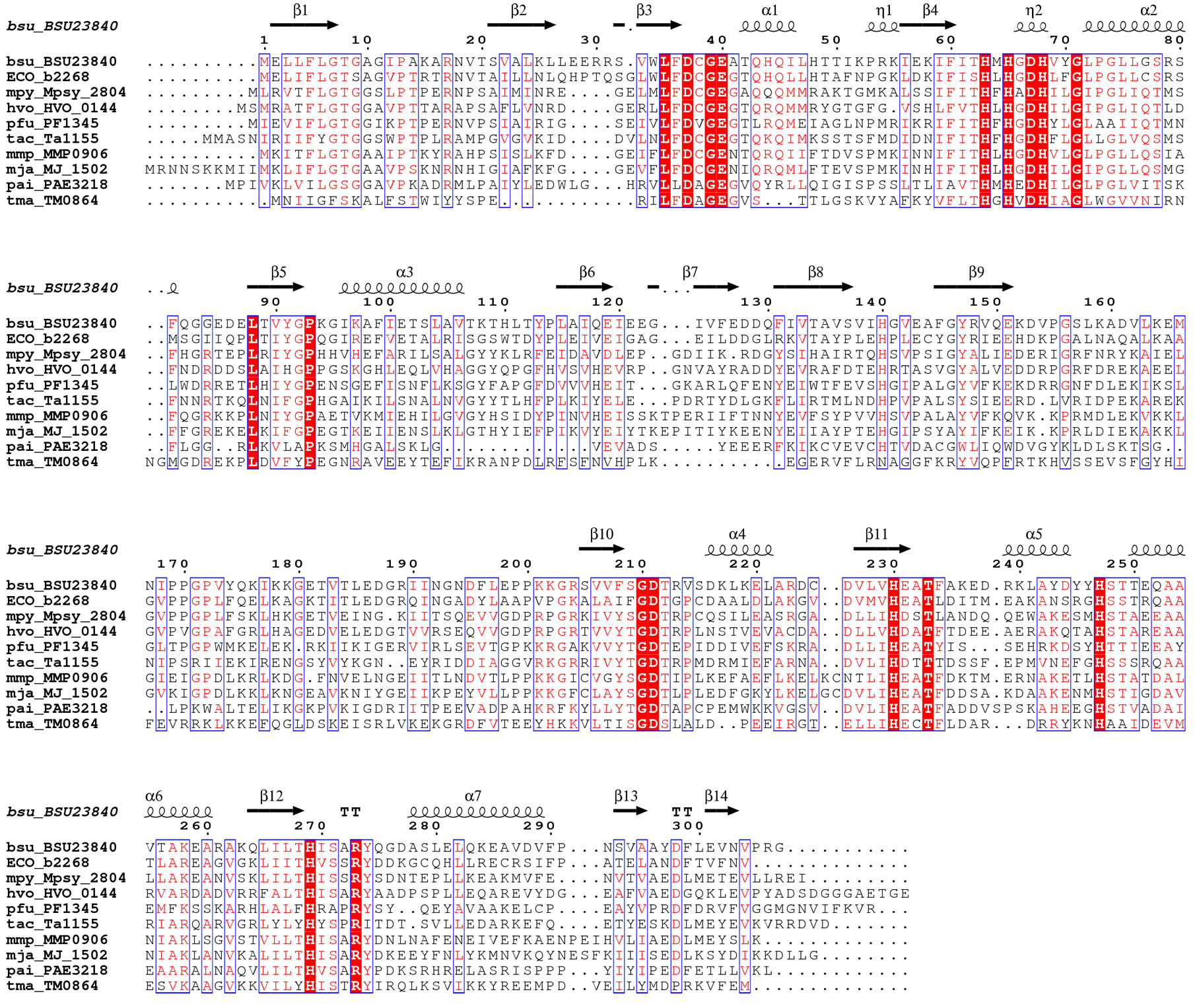

Supplement: FIGURE S1 — Sequence alignment of the RNase Z family proteins. The protein sequences were aligned using ClustalW program (Larkin et al., 2007) and the diagram was prepared using ESPript program (Gouet et al., 1999). Identical residues are highlighted with white type on a red background and similar residues are shown as red type. Methanolobus psychrophilus RNase Z (Mpsy_2804) and Methanococcus maripaludis (MMP0906) shares 38% and 58% amino-acid sequence identity and similarity, respectively. Secondary structural elements of bsu-RNase Z (PDB ID: 1Y44) are shown on the top of the sequence. Bacillus subtilis RNase Z (bsu: Bsu23480) shares 48%, 42%, 42%, 36%, 36%, 32%, 33%, 33%, and 28% amino acid sequence identity to Escherichia coli RNase Z (eco: b2268), M. maripaludis RNase Z (mmp: MMP0906), Methanocaldococcus jannaschii RNase Z (mja: MJ_1502), M. psychrophilus RNase Z (mpy: Mpsy_2804), Haloferax volcanii RNase Z (hvo: HVO_0144), Pyrococcus furiosus RNase Z (pfu: PF12345), Thermoplasma acidophilum RNase Z (tac: Tal155), Pyrobaculum aerophilum RNase Z (pai: PF12345), and Thermotoga maritima RNase Z (tma: TM0864), respectively. [file Image_1.jpg]

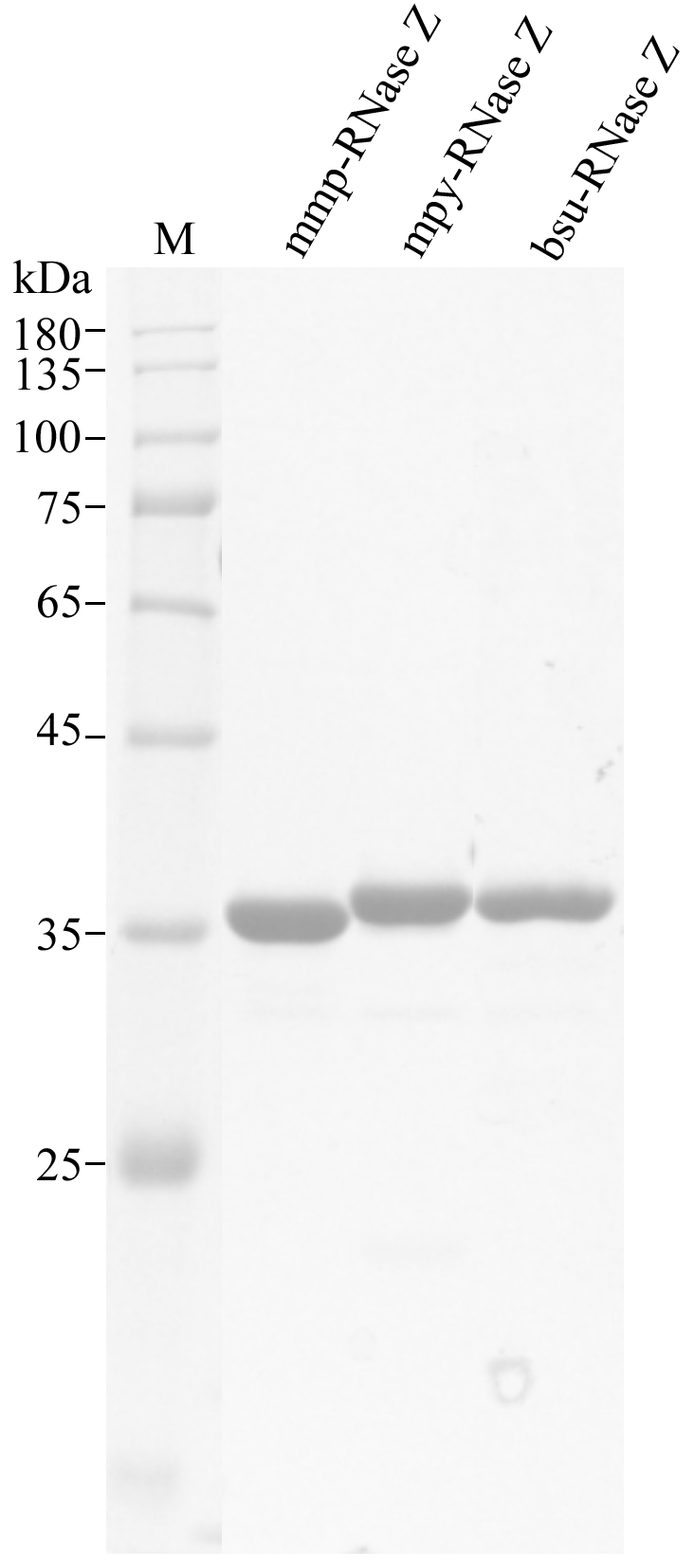

Supplement: FIGURE S2 — SDS-PAGE of the three RNase Zs tested in this study. The purified His6-tagged recombinant RNase Z proteins on 12% SDS-PAGE were shown. M, the protein ladders indicated at the left to identify the migration positions of the proteins. [file Image_2.tif]

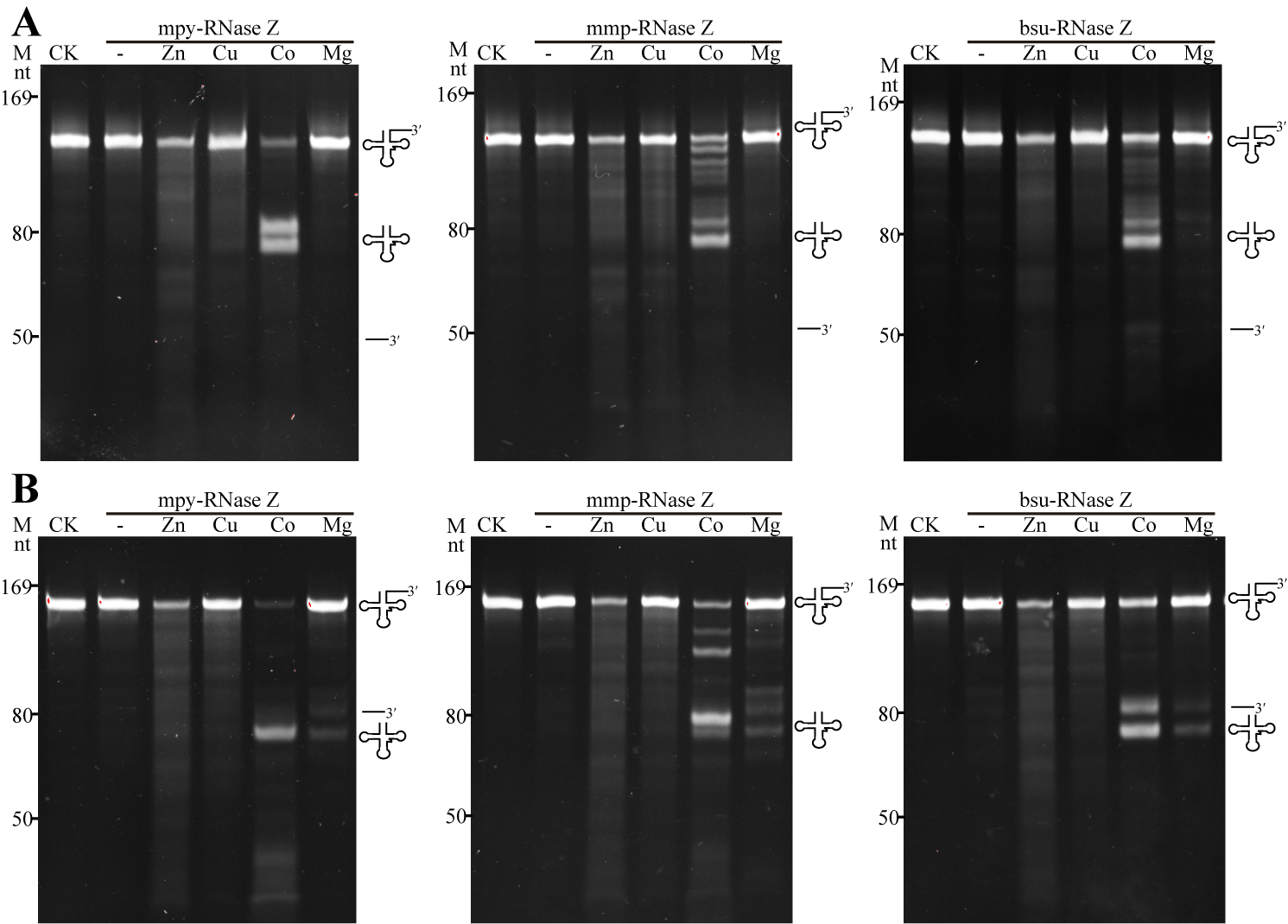

Supplement: FIGURE S3 — Stimulatory effects of Co2+ or Mg2+ on the tRNA 3′-end processing activity of the archaeal and bacterial RNase Zs. Two pre-tRNAs were used as substrates, that is, (A) M. maripaludis S2 pre-tRNAmmp–Arg1 and (B) B. subtilis pre-tRNAbsu–trnI. Purified mpy-RNase Z from M. psychrophilus, mmp-RNase Z from M. maripaludis, and bsu-RNase Z from B. subtilis were assayed in the absence (−) or presence (+) of 1 mM Zn2+, Cu2+, Co2+, or Mg2+. Pre-tRNA (1.4 pmol) was incubated with 0.7 μM mpy-RNase Z, 0.7 μM mmp-RNase Z, and 0.28 μM bsu-RNase Z at 37°C for 30 min. The cleavage products were separated on a 10% polyacrylamide 8 M urea gel. Migration of the ssRNA markers with indicated lengths and migration of the pre-tRNAs, mature tRNAs, and 3′-trailer products are marked at the left and at the right of gels, respectively. [file Image_3.tif]

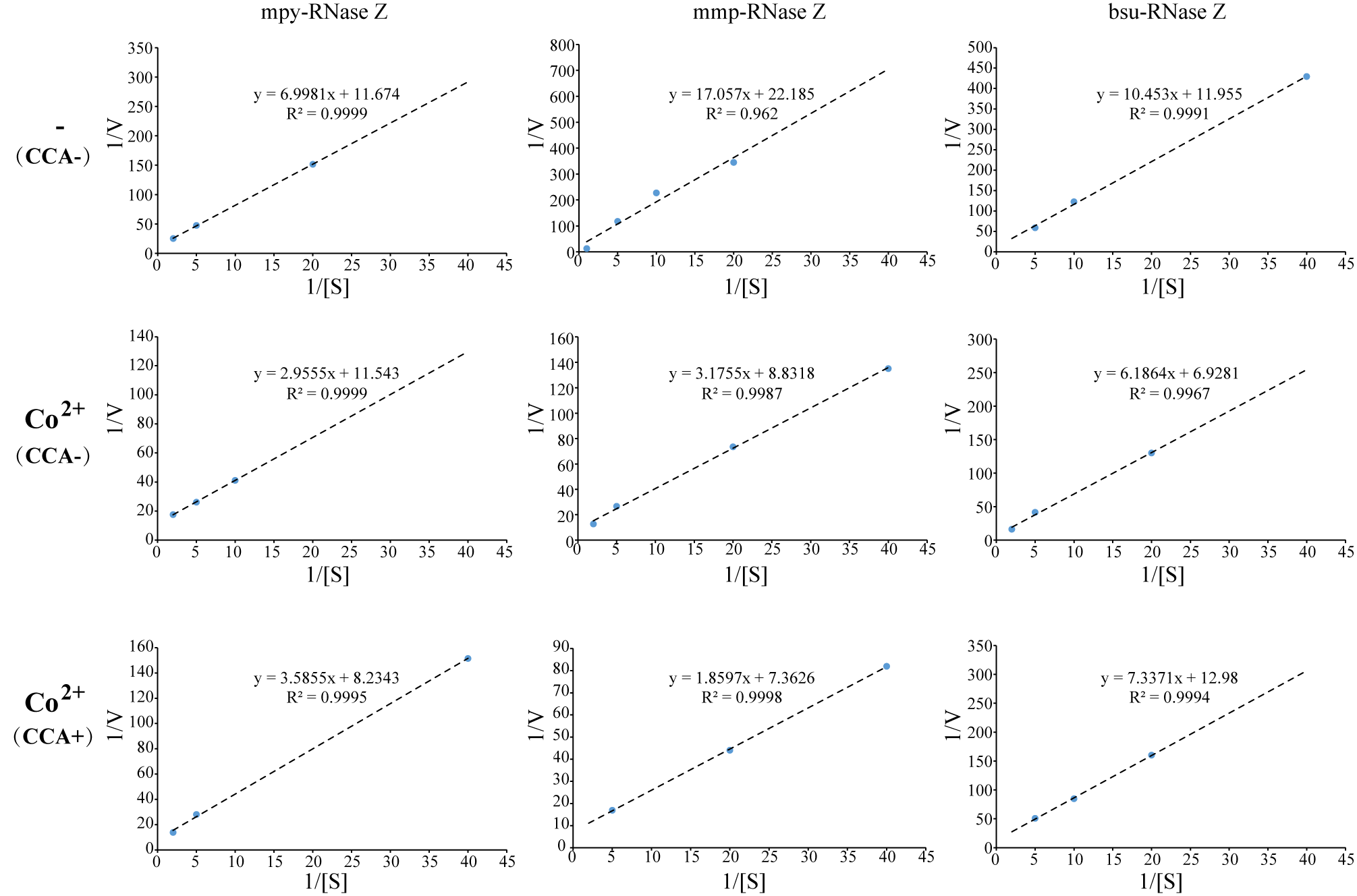

Supplement: FIGURE S4 — The Lineweaver–Burk plots of the kinetic parameters of the three RNase Zs for processing the CCA-containing (CCA+) and CCA-less (CCA−) pre-tRNAs in the absence (−) or presence (+) of Co2+. A range of (0.025–1 μM) pre-tRNA (Table 1) substrate concentrations were assayed for each tested RNase Z at 37°C. The initial velocity (V0) of RNase Z at each substrate concentration was determined through quantifying the substrate residuals in the linear phase during the initial 5 min similarly as that shown in Figure 3. The kinetic parameters of Km, Vmax, and kcat were obtained by fitting the Lineweaver–Burk plotting data to the Michaelis-Menten equation. [file Image_4.tif]

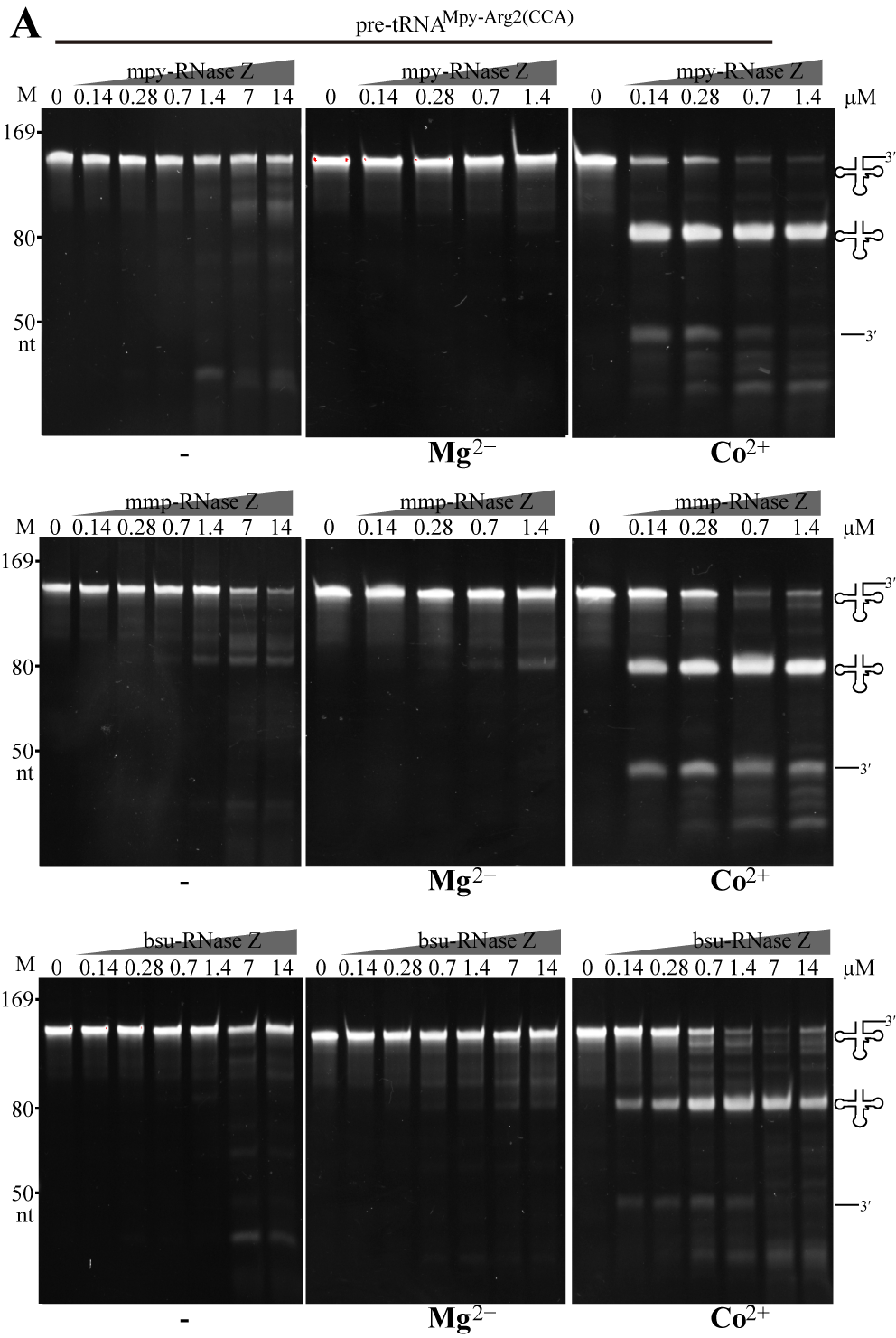

Supplement: FIGURE S5 — Ribonuclease assays of the processing activity of the three RNase Zs on the CCA containing M. psychrophilus pre-tRNAmpy–Arg2(CCA) in the absence (−) or presence of Mg2+ or Co2+. Pre-tRNA (1.4 pmol) was incubated with purified recombinant RNase Z at gradient concentrations in a 10 μl nuclease reaction as described in the “Materials and Methods” section. Cleavage products were separated on a 10% polyacrylamide 8 M urea gel. The migration of ssRNA markers and the migration of pre-tRNAs, mature tRNAs, and 3′-trailer products are labeled at the left and at the right of the gels, respectively. [file Image_5.tif]

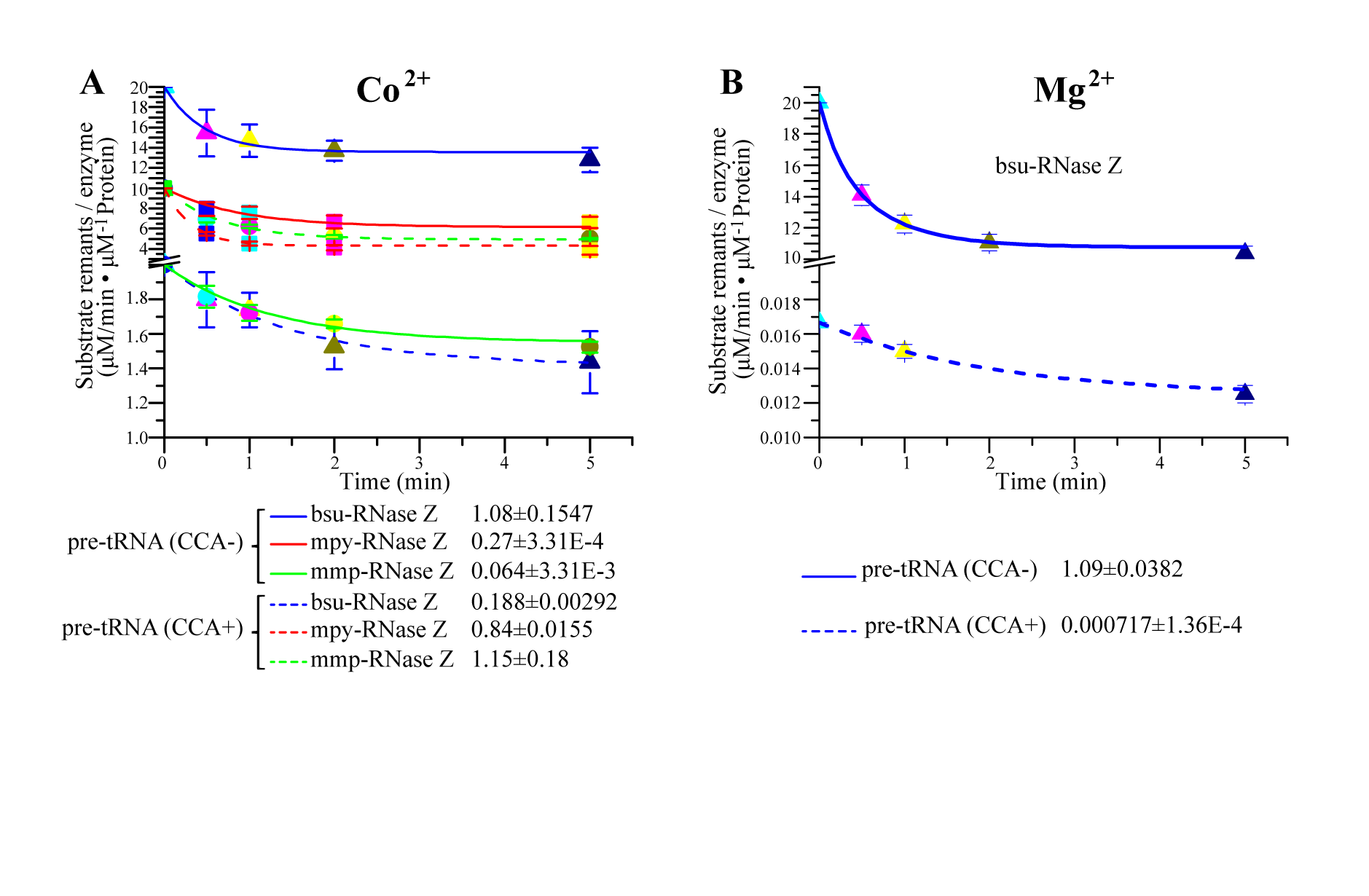

Supplement: FIGURE S6 — Comparison of the initial velocities of the three RNase Zs for processing the CCA-less and CCA-containing pre-tRNAs in Co2+ supplementation (A) and in Mg2+ supplementation for bsu-RNase Z (B). Initial reaction velocities (V0) were determined by quantifying the residual pre-tRNA substrate amounts (listed in Table 1) on PAGE gels at each sampling time, which are shown as attenuation curves of pre-tRNA remnants per the enzyme amounts in the upper panels. Values of V0 (μM/min residual pre-tRNAs⋅μM–1 protein) indicated below the panels are the mean ± s.d from three experimental replicates. [file Image_6.tif]

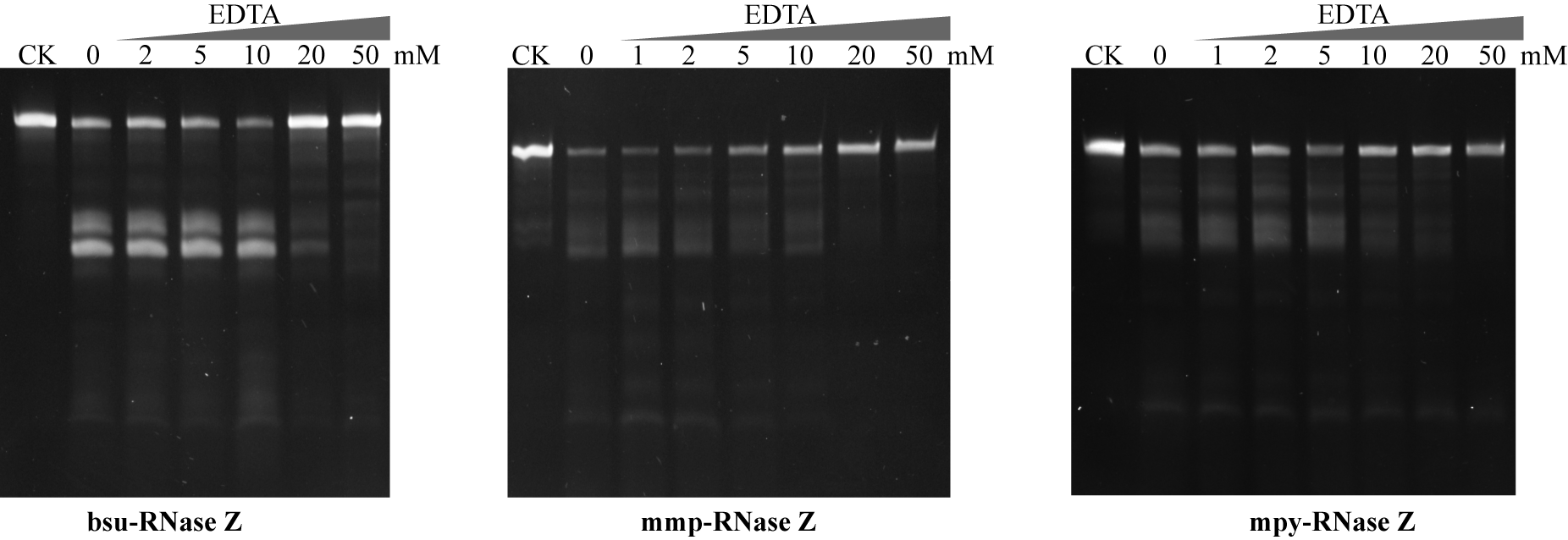

Supplement: FIGURE S7 — EDTA inhibition on the 3′-processing activity of the three RNase Zs. EDTA concentrations were shown on the top of the gel. CK, control reactions without enzyme. [file Image_7.tif]
