# Supplementary material for: Characterization of the Methanomicrobial Archaeal RNase Zs for Processing the CCA-Containing tRNA Precursors
Source: Front Microbiol. 2020 Aug 25;11:1851. doi: 10.3389/fmicb.2020.01851 (PMC7479834; doi:10.3389/fmicb.2020.01851)
Supplement: TABLE S1 — Primers used in this study. [file Table_1.docx]

**Table S1 Primers used in this study.**

| **Primers** | **Sequence (5′→3′)^a^** | **Purpose** |
| --- | --- | --- |
| *Mpsy-*RNase Z-F  *Mpsy-*RNase Z-R | taagaaggagatataccatgcttcgtgtaactttccttg  cagtggtggtggtggtggtgtatttccctgagcaacac | RNaseZ Construction  RNaseZ Construction |
| *Mmp-*RNase Z-F  *Mmp-*RNase Z-R | taagaaggagatataccatgaaaataacttttcttggaacc  cagtggtggtggtggtggtgttttaaagaatattccattaaatcttcc | RNaseZ Construction  RNaseZ Construction |
| *Bsu-*RNase Z-F  *Bsu-*RNase Z-R | taagaaggagatataccatggaattactttttttagggacgggagc  cagtggtggtggtggtggtggcctcgcgggacgtttac | RNaseZ Construction  RNaseZ Construction |
| *Spr-*RNase Z-F  *Spr-*RNase Z-R | ggaattccatatggatattcaatttttaggaacgggggctgg  atatggatccctagatttccacttcttccaagtctttgac | RNaseZ Construction  RNaseZ Construction |
| pET28a-backbone-F  pET28a-backbone-R | caccaccaccaccaccactg  ggtatatctccttcttaaagttaaacaaaattatttctagaggggaattgttatc | Backbone Construction |
| *Mpsy_t8*-F | ccggaattctaatacgactcactataggggagcgtgtggcctagtc | In vitro transcription |
| *Mpsy_t8*-R | ccttattatcaattgtcaatctccg | In vitro transcription |
| *Mpsy_t7*-F  *Mpsy_t7*-R | ccggaattctaatacgactcactatagggggacctgtagtgtagcgga  aatctggatgaagagggc | In vitro transcription  In vitro transcription |
| *Mpsy_t7*-15trailer-F  *Mpsy_t7*-15trailer-R | ccggaattctaatacgactcactatagggggacctgtagtgtagcgga  atgccttttcttttacggac | In vitro transcription  In vitro transcription |
| *Mpsy_t7*-30trailer-F  *Mpsy_t7*-30trailer-R | ccggaattctaatacgactcactatagggggacctgtagtgtagcgga  attttctgcagaattctatgccttttcttttacg | In vitro transcription  In vitro transcription |
| *Mpsy_t7*-150trailer-F  *Mpsy_t7*-150trailer-R | ccggaattctaatacgactcactatagggggacctgtagtgtagcgga  gggaacattacagaaaggaaatgtaag | In vitro transcription  In vitro transcription |
| *Mpsy_t7*-5leader-F  *Mpsy_t7*-5leader-R | ccggaattctaatacgactcactatagggaacacggacctgtagtgtagc  attttctgcagaattctatgccttttcttttacg | In vitro transcription  In vitro transcription |
| *Mpsy_t7*-10leader-F  *Mpsy_t7*-10leader-R | ccggaattctaatacgactcactatagggttttcaacacggacctgtag  attttctgcagaattctatgccttttcttttacg | In vitro transcription  In vitro transcription |
| *Mpsy_t7*-30leader-F  *Mpsy_t7*-30leader-R | ccggaattctaatacgactcactatagggtccatgacacgcccggcaga  attttctgcagaattctatgccttttcttttacg | In vitro transcription  In vitro transcription |
| *Mpsy_t7*-60leader-F  *Mpsy_t7*-60leader-R | ccggaattctaatacgactcactatagggcgacagcagatttattatcaatg  attttctgcagaattctatgccttttcttttacg | In vitro transcription  In vitro transcription |
| *Mpsy_t2*-intron-F  *Mpsy_t2*-intron-R | ccggaattctaatacgactcactataggggggctcgtagggtagccagga  attcaaaaatcctcaaagaaaaagagcaagcg | In vitro transcription  In vitro transcription |
| *Mpsy*_*t15*-intron-F  *Mpsy*_*t15*-intron-R | ccggaattctaatacgactcactatagggctcgccttaactcagtggtag  agcatataaacctttttctggttttgg | In vitro transcription  In vitro transcription |
| *Mpsy_t7*-D arm-del-F  *Mpsy_t7*-D arm-del-R | ccggaattctaatacgactcactatagggggacctgttaagcctccggagc  attttctgcagaattctatgccttttcttttacg | In vitro transcription  In vitro transcription |
| *Mpsy_t7*-anticondon-del-F  *Mpsy_t7*-anticondon-del-R | ccggaattctaatacgactcactatagggggacctgtagtgtagcgga  attttctgcagaattctatgccttttcttttacg | In vitro transcription  In vitro transcription |
| *Mpsy_t7*-T arm-del-F  *Mpsy_t7*-T arm-del-R | ccggaattctaatacgactcactatagggggacctgtagtgtagcgga  attttctgcagaattctatgccttttcttttacg | In vitro transcription  In vitro transcription |
| *Mpsy_t7*-AAacceptor-del-F  *Mpsy_t7*-AAacceptor-del-R | ccggaattctaatacgactcactatagggtagtgtagcggatatcacttaagc  attttctgcagaattctatgccttttc | In vitro transcription  In vitro transcription |
| *Mmp*- *RNA­­_10* -F  *Mmp*- *RNA­­_10* -R | ccggaattctaatacgactcactataggg gggcccgtggcctagtc  tggccggactacaccactgg | In vitro transcription  In vitro transcription |
| *Mmp*- *RNA_14* -F  *Mmp*- *RNA_14* -R | ccggaattctaatacgactcactataggggccctcatggggtagctag  gcaaaacatttgaaaaatgtaaac | In vitro transcription  In vitro transcription |
| *Mmp*- *RNA_14* -15trailer-F  *Mmp*- *RNA_14 -*15trailer-R | ccggaattctaatacgactcactataggggccctcatggggtagctag  aataatataattaaacgcccccatcg | In vitro transcription  In vitro transcription |
| *Mmp*- *RNA_14* -30trailer-F  *Mmp*- *RNA_14* -30trailer-R | ccggaattctaatacgactcactataggggccctcatggggtagctag  aaactttattttaaaaataatataattaaacgcccccatcg | In vitro transcription  In vitro transcription |
| *Mmp*- *RNA_14* -150trailer-F  *Mmp*- *RNA_14* -150trailer-R | ccggaattctaatacgactcactataggggccctcatggggtagctag  gtctattcaataatattaatcttacgtattagttg | In vitro transcription  In vitro transcription |
| *Mmp*- *RNA_14* -5leader-F  *Mmp*- *RNA_14* -5leader-R | ccggaattctaatacgactcactatagggagtatgccctcatggggtag  aaactttattttaaaaataatataattaaacgcccccatcg | In vitro transcription  In vitro transcription |
| *Mmp*- *RNA_14* -10leader-F  *Mmp*- *RNA_14* -10leader-R | ccggaattctaatacgactcactatagggatttgagtatgccctcatgg  aaactttattttaaaaataatataattaaacgcccccatcg | In vitro transcription  In vitro transcription |
| *Mmp*- *RNA_14* -30leader-F  *Mmp*- *RNA_14* -30leader-R | ccggaattctaatacgactcactatagggctcaaaattaagtaattataatttgagtatgc  aaactttattttaaaaataatataattaaacgcccccatcg | In vitro transcription  In vitro transcription |
| *Mmp*- *RNA_14* -60leader-F  *Mmp*- *RNA_14* -60leader-R | ccggaattctaatacgactcactatagggtattttttatgagtatattaaaaaggattactc  aaactttattttaaaaataatataattaaacgcccccatcg | In vitro transcription  In vitro transcription |
| *Mmp*- *RNA_14* -Darm-del-F  *Mmp*- *RNA_14* -Darm-del-R | ccggaattctaatacgactcactataggggccctcatcgcggactgcgga  aaactttattttaaaaataatataattaaacgcccccatcg | In vitro transcription  In vitro transcription |
| *Mmp*-*RNA_14*-anti condon-del-F  *Mmp*-*RNA_14*-anti condon-del-R | ccggaattctaatacgactcactataggggccctcatggggtagctag  aaactttattttaaaaataatataattaaacgcccccatcg | In vitro transcription  In vitro transcription |
| *Mmp*- *RNA_14*-T arm-del-F  *Mmp*- *RNA_14*-T arm-del-R | ccggaattctaatacgactcactataggggccctcatggggtagctag  aaactttattttaaaaataatataattaaacgcccccatcg | In vitro transcription  In vitro transcription |
| *Mmp*-*RNA_14*-AA acceptor-del-F  *Mmp*-*RNA_14-*AA acceptor-del-R | ccggaattctaatacgactcactatagggtggggtagctaggatatcctcgc  aaactttattttaaaaataatataattaaacgcccccatcg | In vitro transcription  In vitro transcription |
| *Bsu_tRNA_24*-83trailer-F  *Bsu_tRNA_24*-83trailer-R | ccggaattctaatacgactcactataggggcttccatagctcagcagg  ggtgttctaccactgaactac | In vitro transcription  In vitro transcription |
| *Bsu_tRNA_53*-47trailer-F  *Bsu_tRNA_53*-47trailer-R | ccggaattctaatacgactcactataggggccggtgtagctcaattg  ctaatggctcttcttacaatagac | In vitro transcription  In vitro transcription |
| *Bsu_tRNA_62*-30trailer -R  *Bsu_tRNA_62*-30trailer -R | ccggaattctaatacgactcactatagggggacctttagctcagttggttag  tcagccagacttgggtattcct | In vitro transcription  In vitro transcription |
| *Mmp*- *RNA­­_10-* RACE -F  *Mmp*- *RNA­­_10-* RACE -R | ccggaattctaatacgactcactataggg gggcccgtggcctagtc  ctgtaggcaccatcaat | 3′RACE  3′RACE |
| *Mmp*- *RNA_14-* RACE -F  *Mmp*- *RNA_14-* RACE -R | ccggaattctaatacgactcactataggggccctcatggggtagctag  ctgtaggcaccatcaat | 3′RACE  3′RACE |

1. The T7 promoter sequence for in vitro transcription is underlined.
