# Supplementary material for: Characterization of the Methanomicrobial Archaeal RNase Zs for Processing the CCA-Containing tRNA Precursors
Source: Front Microbiol. 2020 Aug 25;11:1851. doi: 10.3389/fmicb.2020.01851 (PMC7479834; doi:10.3389/fmicb.2020.01851)
Supplement: TABLE S2 — Sequences of RNA substrates used in this study. [file Table_2.docx]

**Table S2** **Sequences of RNA substrates used in this study.**

| **Name** | **Sequence ^a^** |
| --- | --- |
| **pre_tRNA*^mpy^*^-Arg2(CCA)^**  (*Mpsy_t8*) | 5′gagcguguggccuagucaggauauggcggcagccuccuaagcugcaagccgaggguucaaaucccuucacgcucgccauuuucucuuuuuagaacaaucacaacggagauugacaauugauaauaagg3′ |
|  |  |
| **pre_tRNA*^mpy^*^-Arg1^**  (*Mpsy_t7*) | 5′ggaccuguaguguagcggauaucacuuaagccuccggagcuuagaacccggguucgagucccggcagguccguaaaagaaaaggcauagaauucugcagaaaaugcccucuucauccagauu3′ |
|  |  |
| **pre_tRNA*^mpy^*^-Arg3(intron)^**  (*Mpsy_t2*) | 5′gggcucguaggguagccaggauauccugaugggcuucgaagagguuuuauuugccccgaagacacccauugacccgcguucgaaucgcggcgggcccgcuugcucuuuuucuuugaggauuuuugaau3′ |
|  |  |
| **pre_tRNA*^mpy^*^-Tyr(intron)^**  (*Mpsy_t15*) | 5′cucgccuuaacucagugguagagugcgcggcuguaguaugguucaugugacuuguucacacauaccgcgcaggcaccgcgauguccccgguucgagucugggaggcgggacuuccaaaaccagaaaaagguuuauaugcu3′ |
|  |  |
| **pre_tRNA*^mpy^*^-Arg1^**  (*Mpsy_t7*)  D arm-del | 5′ggaccuguuaagccuccggagcuuagaacccggguucgagucccggcagguccguaaaagaaaaggcauagaauucugcagaaaau3′ |
|  |  |
| **pre_tRNA*^mpy^*^-Arg1^**  (*Mpsy_t7*)  anti condon-del | 5′ggaccuguaguguagcggauaucacugaacccggguucgagucccggcagguccguaaaagaaaaggcauagaauucugcagaaaau3′ |
|  |  |
| **pre_tRNA*^mpy^*^-Arg1^**  (*Mpsy_t7*)  Darm-del | 5′ggaccuguaguguagcggauaucacuuaagccuccggagcuuagaaccagguccguaaaagaaaaggcauagaauucugcagaaaau3′ |
|  |  |
| **pre_tRNA*^mpy^*^-Arg1^**  (*Mpsy_t7*)  AA accepuor-del | 5′uaguguagcggauaucacuuaagccuccggagcuuagaacccggguucgagucccgguaaaagaaaaggcauagaauucugcagaaaau3′ |
|  |  |
| **pre_tRNA*^mmp^*^-Arg2(CCA)^**  *RNA­­_10* | 5′gggcccguggccuagucuggauacggcaccggccuucuaagccggggaucggggguucgaaucccuccggguccgccauuacuacuuuucaagauuuaugcaaaugcuccagugguguaguccggcca3′ |
| **pre_tRNA*^mmp^*^-Arg1^**  *RNA_14* | 5′gcccucaugggguagcuaggauauccucgcggacugcggauccguggacucggguucaaaucccgaugggggcguuuaauuauauuauuuuuaaaauaaaguuuacauuuuucaaauguuuugc3′ |
| **pre_tRNA*^mmp^*^-Arg1^**  *RNA_14*  D arm-del | 5′gcccucaucgcggacugcggauccguggacucggguucaaaucccgaugggggcguuuaauuauauuauuuuuaaaauaaaguuu3′ |
| **pre_tRNA*^mmp^*^-Arg1^**  *RNA_14*  anti condon-del | 5′gcccucaugggguagcuaggauauccugacucggguucaaaucccgaugggggcguuuaauuauauuauuuuuaaaauaaaguuu3′ |
| **pre_tRNA*^mmp^*^-Arg1^**  *RNA_14*  D arm-del | 5′gcccucaugggguagcuaggauauccucgcggacugcggauccguggacugggggcguuuaauuauauuauuuuuaaaauaaaguuu3′ |
| **pre_tRNA*^mmp^*^-Arg1^**  *RNA_14*  AA accepuor -del | 5′ugggguagcuaggauauccucgcggacugcggauccguggacucggguucaaaucccgauuuaauuauauuauuuuuaaaauaaaguuu3′ |
| **pre_tRNA*^Bsu^*^-trnI^**  *Bsu_tRNA_24* | 5′gcuuccauagcucagcagguagagcacuuccaugguaaggaagaggucagcgguucgagcccgcuuggaagcuuaaauguauuauuaccaagguuucucauaaggagaaagcuuuuuuuauugcgauaugcggaaguaguucagugguagaacacc3′ |
| **pre_tRNA*^Bsu^*^-trnB(CCA)^**  *Bsu_tRNA_53* | 5′gccgguguagcucaauugguagagcaacugacuuguaaucaguagguuggggguucaaguccucuugccggcaccacuuuuauaugauauaauauucaagucuauuguaagaagagccauuag3′ |
| **pre_tRNA*^Bsu^*^-t62(CCA)^**  *Bsu_tRNA_62* | 5′ggaccuuuagcucaguugguuagagcagacggcucauaaccguccggucguagguucgaguccuacaagguccaccacuauacggaggaauacccaagucuggcuga3′ |
| **pre_tRNA*^Soi^*^-Arg^**  *I872_t10790* | 5′ggucucauagcucagcuggauagagcauucgccuucuaagcgaacggucgcagguucgaauccugcugggaucauaauauacacuacucagacacgaaaaauuu3′ |
|  |  |

1. The 3′ trailer sequence is underlined.
